# uPAR/suPAR Signaling and Organ Crosstalk in Cardiovascular-Kidney-Metabolic Syndrome

**DOI:** 10.1161/CIRCRESAHA.126.328563

**Published:** 2026-06-19

**Authors:** Salim S. Hayek, Stuart E. Dryer

**Affiliations:** Department of Internal Medicine, University of Texas Medical Branch, Galveston, TX (S.S.H.).; Division of Cardiology, University of Michigan, Ann Arbor, MI (S.S.H.).; Department of Biology and Biochemistry, University of Houston, TX (S.E.D.).

**Keywords:** atherosclerosis, chronic kidney disease, diabetes mellitus, type 2, fibrosis, heart failure, inflammation, podocytes

## Abstract

Cardiovascular-kidney-metabolic syndrome is driven by inflammatory mechanisms that propagate injury across organ boundaries, yet the molecular mediators converting systemic inflammation into end-organ damage remain incompletely defined. Across multiple prospective cohorts, suPAR (soluble urokinase plasminogen activator receptor) is associated with incident cardiovascular events, heart failure, diabetes, and kidney disease progression, and genetic and experimental studies support a causal role. How a glycosylphosphatidylinositol-anchored receptor without a transmembrane domain initiates intracellular signaling has remained a fundamental paradox. A membrane-tethered receptor, uPAR, addresses this paradox by assembling lateral signalosomes with coreceptors including αvβ3 integrin, the receptor for advanced glycation end-products, and receptor tyrosine kinases on myeloid, endothelial, and vascular smooth muscle cells, transducing signals that drive atherosclerotic plaque inflammation, vascular remodeling, and maladaptive fibrosis. Proteolytic and lipolytic cleavage of uPAR releases suPAR and its fragments into the circulation; a cleavage/release switch that converts locally scaffolded signaling into diffuse systemic agonist activity and links inflammation in 1 tissue to injury in distant organs. SuPAR activates podocyte αvβ3 integrin and receptor for advanced glycation end-products, now identified as an obligate coreceptor, triggering a Rac1/NOX2–Src–TRPC6 (transient receptor potential canonical channel 6) cascade that produces proteinuria and glomerulosclerosis. The D2D3 cleavage fragment drives insulin-dependent diabetes in transgenic mice through direct β-cell toxicity, an effect reversed by anti-uPAR antibody. This framework reframes the uPAR/suPAR axis not as a single biomarker but as a compartmentalized signaling system operating in distinct modes across cardiovascular-kidney-metabolic-relevant cell types. We map pharmacologically tractable intervention nodes spanning transcriptional suppression, suPAR neutralization, receptor interface disruption, and downstream kinase and channel inhibition, and propose that matching therapeutic strategy to the predominant signaling mode may enable disease-context-dependent precision approaches to cardiovascular-kidney-metabolic syndrome.

Cardiovascular-kidney-metabolic (CKM) syndrome describes shared inflammatory mechanisms that drive injury across cardiovascular, renal, and metabolic specialty boundaries. A central challenge is understanding how inflammation originating in 1 compartment (bone marrow, adipose tissue, liver) causes pathology in distant organs. Traditional risk factors incompletely explain this crosstalk; patients with well-controlled glucose, lipids, and blood pressure still progress, implying additional mediators bridge systemic inflammation and end-organ damage.^1,2^

## uPAR and suPAR: From Fibrinolytic Receptor to CKM Mediator

How does a receptor lacking transmembrane and cytoplasmic domains initiate intracellular signaling cascades that drive this multiorgan pathology? The uPAR (urokinase-type plasminogen activator receptor/CD87) and its suPAR (soluble uPAR) have emerged as key components of the inflammatory bridge linking CKM disease manifestations. Originally characterized as a glycosylphosphatidylinositol-anchored receptor that localizes urokinase-mediated plasminogen activation to the cell surface, uPAR is now recognized as a versatile signaling scaffold that assembles multi-protein complexes with integrins, RTKs (receptor tyrosine kinases), GPCRs (G protein-coupled receptors), and the RAGE (receptor for advanced glycation end-products) to transduce extracellular cues into intracellular responses.^3–8^ Its localization within cholesterol-rich lipid rafts facilitates rapid activation of Src family kinases and other transducing proteins enriched in these membrane nanodomains.^9^ Because uPAR lacks transmembrane and cytoplasmic domains, it achieves signaling competence only through dynamic coreceptor assembly.

The significance of this axis extends well beyond a signaling curiosity. Elevated suPAR independently predicts incident cardiovascular disease, coronary artery calcification, heart failure, chronic kidney disease (CKD) progression, acute kidney injury, incident type 2 diabetes, and all-cause mortality across diverse populations.^1,2,10,11^ Because observational associations alone cannot distinguish causation from confounding, Mendelian randomization analyses of *PLAUR* variants (notably rs4760) provide stronger causal evidence, subject to standard assumptions including absence of horizontal pleiotropy.^11^ Complementary experimental evidence substantially strengthens the causal inference: in transgenic mice overexpressing suPAR, atherosclerosis is accelerated through enhanced monocyte chemotaxis and plaque inflammation despite equivalent cholesterol levels, and a cleaved suPAR fragment (D2D3) causes both kidney disease and pancreatic β-cell dysfunction, effects reversed by an anti-uPAR antibody.^12^ Genetic deletion or antibody-mediated neutralization of suPAR also ameliorates kidney injury in experimental models.^13,14^ Together, the convergence of epidemiological predictions, genetic causal inference, and interventional experimental evidence establishes that the uPAR/suPAR axis is not merely a biomarker of inflammation but an active mediator linking immune activation to cardiovascular, kidney, and metabolic injury: the defining triad of CKM syndrome.

## A Membrane-Tethered Versus Soluble Signaling Framework

**Figure 1.**
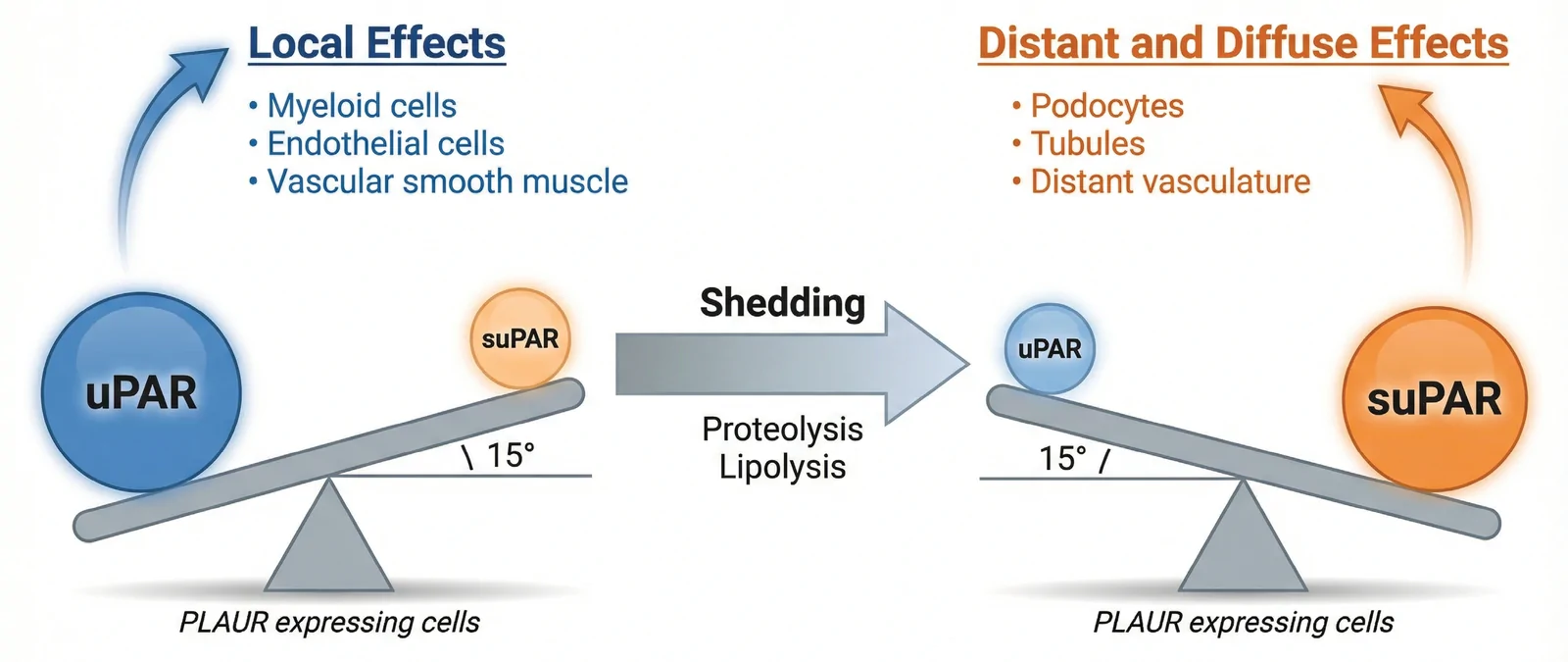
**The uPAR (urokinase-type plasminogen activator receptor)/suPAR (soluble urokinase plasminogen activator receptor) signaling axis: local vs diffuse modes of action.** Membrane-bound, glycosylphosphatidylinositol (GPI)-tethered uPAR (PLAUR [plasminogen activator, urokinase receptor]) binds uPA and assembles coreceptor signalosomes to drive localized adhesion, migration, and remodeling in myeloid, endothelial, and vascular smooth muscle cells. Proteolytic or lipolytic release generates suPAR (full-length D1–D2–D3 and cleaved D2–D3) that engages distant receptor complexes (eg, αvβ3 integrin, receptor for advanced glycation end-products [RAGE]) on target cells, including podocytes, tubular epithelium, and vasculature, promoting CKM syndrome progression and highlighting diffuse suPAR signaling as a therapeutic target.

These observations point to a receptor system that operates in 2 fundamentally distinct modes. In its membrane-tethered form, uPAR functions as a cell-local signaling scaffold, assembling coreceptor signalosomes that drive adhesion, migration, and tissue remodeling on myeloid cells, endothelial cells, and VSMC (vascular smooth muscle cells). Proteolytic or lipolytic cleavage of the glycosylphosphatidylinositol anchor releases suPAR into the systemic circulation, converting a membrane-tethered receptor into a circulating agonist that engages coreceptors, including αvβ3 integrin and RAGE, on distant target cells such as podocytes, TEC (tubular epithelial cells), and vascular endothelium.^8,15,16^ This cleavage/release switch attenuates local uPAR-dependent signaling by depleting surface receptors, while simultaneously broadcasting inflammatory signals systemically.^1,2,10,17^ These modes are heuristic anchor points on a continuum: suPAR can act in paracrine loops before entering the circulation, and membrane uPAR contributes to multicellular programs via cell–cell interactions,^18–20^ analogous to insulin or glucagon, whose paracrine effects do not invalidate their endocrine framework. The value of the local-versus-diffuse distinction is not that it draws an absolute spatial boundary but that it organizes therapeutic strategy: interventions targeting membrane uPAR address the signaling scaffold at its source, whereas interventions targeting circulating suPAR intercept the soluble mediator en route to distant organs. This framework resolves apparent contradictions in the literature regarding when the uPAR/suPAR axis promotes local tissue remodeling versus remote organ damage (Figure 1).

In this review, we address how uPAR achieves pathway specificity through signalosome assembly despite lacking intrinsic signaling domains; what molecular mechanisms distinguish local uPAR signaling from soluble suPAR signaling that causes remote organ injury; and which signaling nodes offer tractable therapeutic targets in CKM syndrome. We integrate structural biology, biophysical mechanisms, and cell-type-specific signaling programs, focusing on pathways with established relevance to atherosclerosis, diabetic kidney disease, acute kidney injury, and metabolic inflammation. This framework builds on studies cataloguing uPAR-interacting proteins and the prognostic value of suPAR,^6,21^ and is motivated by recent discoveries, including the identification of RAGE as a suPAR coreceptor,^14,22^ the finding that formyl peptide receptors form complexes with β3-integrin subunits and mediate β-arrestin-dependent suPAR signaling,^23^ and the recognition that Factor XII engages uPAR through a distinct integrin β1-dependent axis in diabetic kidney disease.^24^

## Structural and Biophysical Determinants of Signaling Competence

### Domain Architecture of uPAR and Its Conformational Dynamics

**Figure 2.**
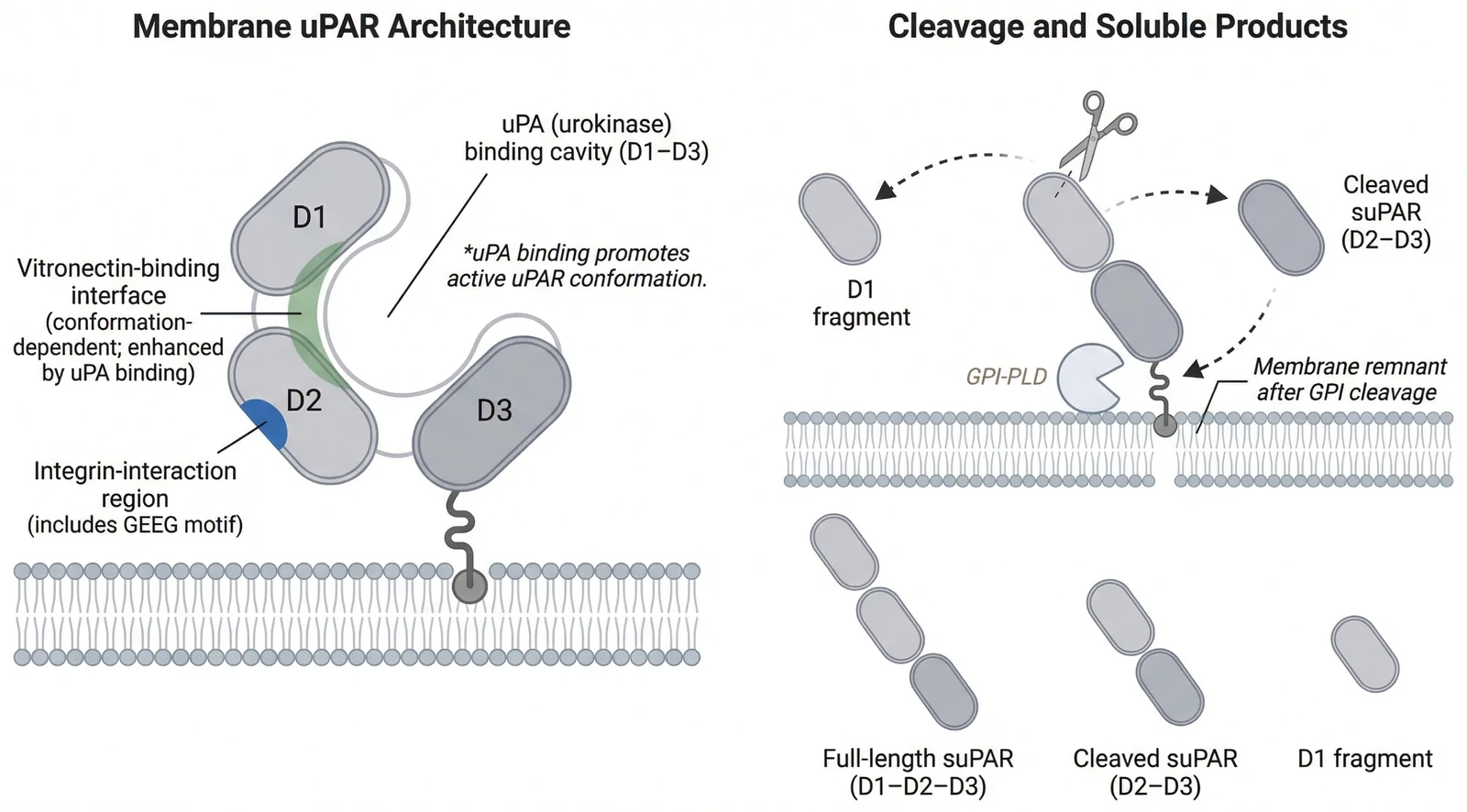
**uPAR (uPA [urokinase-type plasminogen activator] receptor) structure and generation of soluble suPAR (soluble urokinase plasminogen activator receptor) species. A**, uPAR domain organization (D1–D3) as a glycosylphosphatidylinositol (GPI)-anchored membrane receptor with a central uPA-binding cavity and external interaction surfaces. **B**, Conceptual uPAR conformational states (ligand-free vs uPA-stabilized). **C**, Shedding/proteolysis generates circulating full-length suPAR and cleaved fragments (D2–D3 and D1). GEEG indicates Gly Glu-Glu-Gly.

uPAR comprises 3 consecutive Ly6/uPAR domains (D1, D2, D3), each containing ≈90 amino acids, stabilized by multiple disulfide bonds characteristic of the 3-finger fold^25–28^ (Figure 2). Crystal structures reveal a semi-circular configuration that generates both a deep central cavity for urokinase binding and an extensive external surface available for coreceptor interactions. However, static crystal structures underrepresent the conformational dynamics essential for uPAR function.

Solution-phase studies (small-angle X-ray scattering, hydrogen-deuterium exchange mass spectrometry, molecular dynamics) show that ligand-free uPAR is flexible, with D1 exhibiting conformational freedom relative to the more rigid D2–D3 unit.^26,29^ This flexibility reflects a missing consensus disulfide bond found in canonical Ly6/uPAR domains; its experimental introduction constrains the receptor and impairs both urokinase binding and the interdomain rearrangements required for signaling.^29^

Ligand binding converts this flexibility into defined signaling-competent states. Urokinase binding compacts the 3-domain structure, stabilizing D1 and reducing its conformational entropy.^26,29^ This conformational clamp enhances uPAR affinity for vitronectin and increases integrin activation through a tension-dependent mechanism: uPAR-vitronectin engagement generates lateral membrane forces transmitted to associated integrins, triggering their activation even without direct integrin-matrix contact.^30–33^ Conversely, artificially constraining uPAR through a non-native D1–D3 disulfide bond drives constitutive lamellipodia formation independent of urokinase, confirming that conformational state, not just ligand occupancy, determines signaling output.^32^ These observations point to biased signaling analogous to GPCRs: different ligands stabilize distinct uPAR conformations that select among coreceptors laterally rather than through intracellular conformational coupling.^26,29^ A structural basis for this selectivity lies in D2, which contains an integrin-interacting epitope centered on a GEEG (Gly Glu-Glu-Gly) sequence that directly contacts αvβ3 integrin.^25,34^ Synthetic peptides corresponding to this region activate αvβ3-dependent signaling, while sequence-altered peptides (Gly Ala-Ala-Gly) function as inhibitors, making this epitope a tractable target for disrupting pathological uPAR–integrin interactions while preserving fibrinolytic function.^34^

### Cleavage, Release, and Generation of suPAR Isoforms

**Figure 3.**
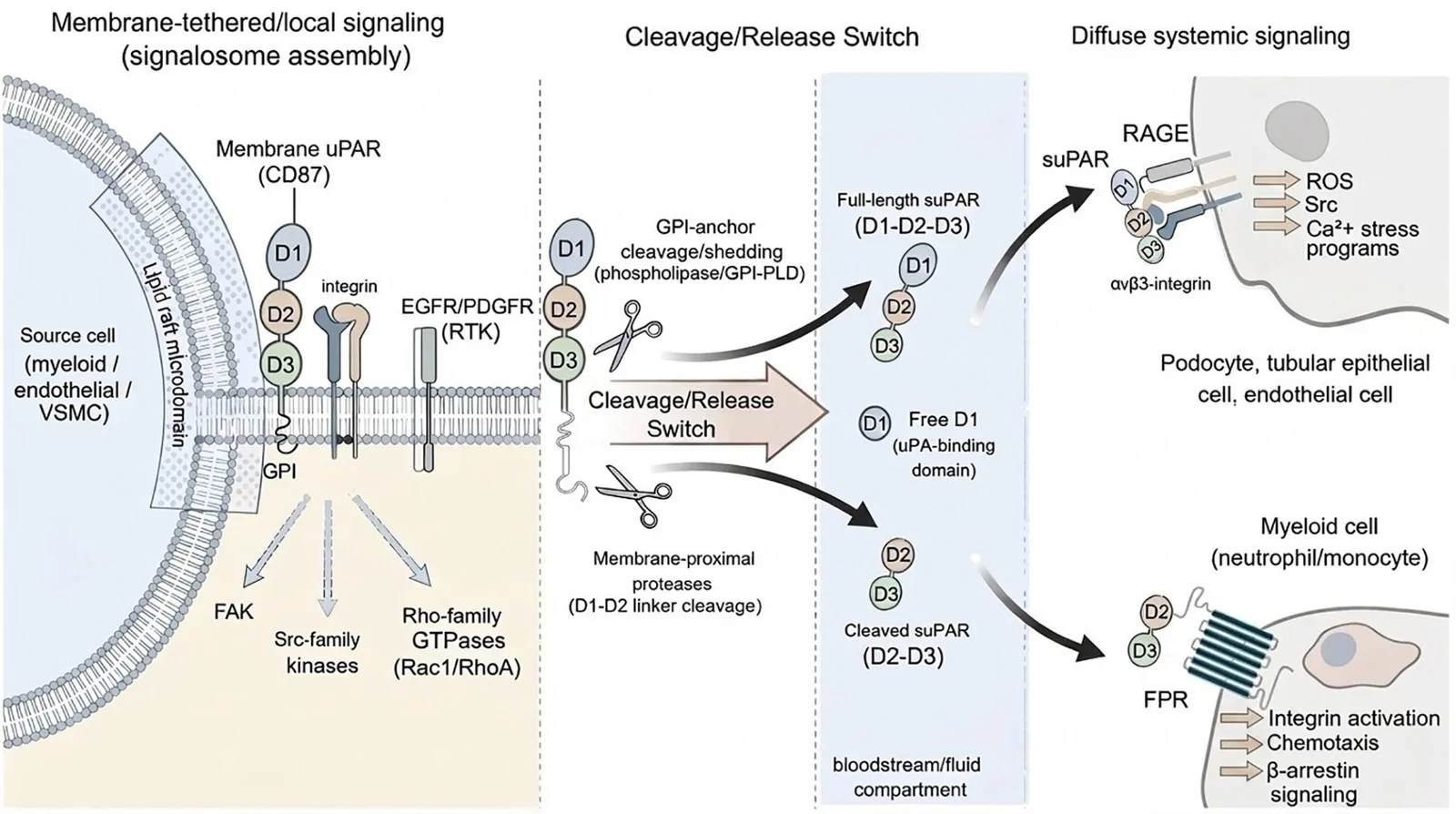
**Local vs diffuse signaling framework of the uPAR (urokinase-type plasminogen activator receptor)/suPAR (soluble urokinase plasminogen activator receptor) axis.** Membrane uPAR supports local, cell-surface signaling through coreceptor assembly, whereas released suPAR mediates diffuse, systemic signaling on distant target cells; the cleavage/release switch redirects signaling reach. EGFR indicates epidermal growth factor receptor; FAK, focal adhesion kinase; FPR, formyl peptide receptor; GPI, glycosylphosphatidylinositol; PDGFR, platelet-derived growth factor receptor; PLD, glycosylphosphatidylinositol-specific phospholipase D; RAGE, receptor for advanced glycation end-products; ROS, reactive oxygen species; RTK, receptor tyrosine kinase; uPA, urokinase-type plasminogen activator; and VSMC, vascular smooth muscle cell.

The transition from local to systemic signaling begins when uPAR is shed from the cell surface. Three principal mechanisms release the receptor, none involving classical intracellular phospholipases. (1) GPLD1 (glycosylphosphatidylinositol-specific phospholipase D), a secreted HDL-associated enzyme, cleaves the glycosylphosphatidylinositol anchor directly; overexpression increases suPAR shedding, and knockdown reduces it.^35^ GPLD1 concentrates in atherosclerotic plaques, colocalizing with macrophages and oxidation epitopes,^36^ and its serum levels correlate with insulin resistance and dyslipidemia,^37^ suggesting metabolic enhancement of glycosylphosphatidylinositol-mediated release. (2) The transmembrane glycerophosphodiester phosphodiesterase GDE3 (GDPD5) functions as a glycosylphosphatidylinositol-specific phospholipase C with its catalytic domain oriented extracellularly; it cleaves the uPAR glycosylphosphatidylinositol anchor and releases functional suPAR, whereas the homologous GDE2 cannot, demonstrating substrate specificity.^38^ Membrane-proximal proteases, including uPA itself, MMPs (matrix metalloproteinases), and cathepsin G, cut within or near the glycosylphosphatidylinositol-attachment site to release full-length soluble uPAR (suPAR I–III, residues 1–283) containing all 3 Ly6/uPAR domains.^35,39–41^ In activated neutrophils, proteolytic shedding predominates and generates the truncated D2D3 form, enhanced by calcium influx and FPR (formyl peptide receptor) agonists.^42^ The relative contributions of lipolytic and proteolytic shedding are cell-type- and context-dependent. Secondary cleavage within the D1–D2 linker, mediated by uPA, plasmin, or other serine proteases, liberates D2D3 and frees D1 as a separate species (Figure 3).

Full-length suPAR and D2D3 are not interchangeable. Full-length suPAR retains the capacity to bind urokinase and vitronectin and can engage αvβ3 integrin on distant target cells, recapitulating membrane uPAR signaling in a paracrine or endocrine mode. D2D3, by contrast, loses the uPA-binding cavity (which requires D1) but exposes a chemotactic epitope that potently activates FPRs on myeloid cells, driving integrin activation, chemotaxis, and β-arrestin-dependent signaling.^8^ Both species are detectable in the circulation, although full-length suPAR predominates in most clinical assays.^43^

Shedding has dual consequences central to this review’s framework. Depletion of surface uPAR attenuates local adhesion-dependent processes, while released species broadcast inflammatory signals systemically: full-length suPAR through integrin/RAGE engagement, D2D3 through FPR-dependent myeloid recruitment (Figure 3). Excessive proteolysis at inflamed sites thus attenuates local migration while amplifying systemic signaling, helping explain why suPAR rises in proportion to disease severity across CKM conditions. Inflammatory cytokines (TNF-α, IL-1β [interleukin-1β]), growth factors, and integrin engagement modulate shedding in a stimulus- and cell-type-dependent manner.^6,44^

### Lipid Raft Compartmentalization and uPAR Membrane Dynamics

The glycosylphosphatidylinositol anchor targets uPAR to cholesterol-rich lipid rafts and caveolae, membrane nanodomains that concentrate signaling proteins including Src-family kinases, integrins, and caveolin.^3,45,46^ The functional significance of this compartmentalization is clear: cholesterol depletion disrupts lipid raft integrity and impairs both uPAR-dependent signaling and pericellular plasminogen activation.^47^ Within raft domains, uPAR exhibits high lateral mobility as demonstrated by fluorescence recovery after photobleaching and single-particle tracking,^47^ enabling dynamic sampling of the nanodomain for transient or stable association with integrins and other signaling partners. The resulting search mechanism contrasts with most transmembrane receptors and may explain how uPAR, despite typically modest surface expression, efficiently catalyzes signalosome assembly. Recent uPAR-dimer crystal structures suggest an additional regulatory layer: D1 opens into an expanded ring capturing a β-hairpin from a neighboring uPAR molecule, potentially preorganizing signaling complexes and lowering activation thresholds.^28^

## uPAR as a Membrane Signaling Scaffold

### Integrin Subtypes as uPAR Coreceptors

Integrins are the canonical signaling partners for membrane-tethered uPAR, with extensive evidence demonstrating direct physical interactions and functional crosstalk.^20,30,48,49^ Three integrin subfamilies are particularly relevant to CKM syndrome.

β1 integrins (α3β1, α5β1) mediate cell adhesion to fibronectin and laminin and regulate tissue fibrosis and VSMC function. uPAR binds to the β-propeller domain of α5β1, converting the integrin into an Arg-Gly Asp-resistant high-affinity state independent of extracellular matrix ligand engagement.^20^ In atherosclerosis, β1 integrin activation in VSMCs promotes proliferation and migration, key events in neointimal hyperplasia and plaque progression.^50,51^ The uPAR-β1 axis may therefore contribute to adverse vascular remodeling after percutaneous coronary intervention, where both uPAR expression and integrin-dependent VSMC migration are upregulated.^52^

β2 integrins (αMβ2/Mac-1 [macrophage-1 antigen], αLβ2/LFA-1 [lymphocyte function-associated antigen-1]) are restricted to leukocytes and mediate immune cell adhesion and arrest on inflamed endothelium. The uPAR–Mac-1 interaction is bidirectional: uPAR serves as a counter-receptor for Mac-1, promoting monocyte adhesion to endothelium, while uPAR engagement also modulates Mac-1 affinity for fibrinogen and ICAM-1 (intercellular adhesion molecule 1) through inside-out signaling.^53–55^ This bidirectional regulation positions uPAR as a coordinator of leukocyte recruitment in atherosclerosis.

β3 integrins (αvβ3, αIIbβ3) are expressed on platelets, endothelial cells, and podocytes. Structure-function studies demonstrate that uPAR binds integrin α-subunit β-propeller domains via D3, inducing conformational changes in the integrin headpiece that favor its high-affinity open state.^32,48^ Vitronectin, enriched in provisional matrices and atherosclerotic plaques, interacts directly with uPAR and anchors cells to the extracellular matrix.^31,56^ The uPAR-vitronectin interaction drives allosteric changes in αvβ3 and αIIbβ3 integrins, activating FAK (focal adhesion kinase) and downstream cascades even without direct integrin–vitronectin contact.^57^ Alanine-scanning mutagenesis has identified a surface epitope on uPAR required for vitronectin binding; disruption of this interface abolishes uPAR-dependent adhesion, migration, and signal transduction.^31,58^ The vitronectin-binding site overlaps with the SMB (somatomedin B) domain that also binds PAI-1 (plasminogen activator inhibitor-1). Consequently, PAI-1 competitively disrupts uPAR-vitronectin interactions, functioning as a negative regulator of uPAR-dependent adhesion, a mechanism with direct CKM relevance given that PAI-1 levels are elevated in metabolic syndrome and type 2 diabetes.^59,60^

### RTK Transactivation

uPAR extends its signaling reach beyond integrins through transactivation of RTKs, particularly the EGFR (epidermal growth factor receptor) and PDGFR-β (platelet-derived growth factor receptor-β).^5,52,61^ uPAR and EGFR colocalize at the cell surface and coimmunoprecipitate, suggesting ternary complex formation in which uPAR scaffolds integrin–RTK assemblies within lipid raft microdomains.^62–64^ uPAR knockdown alters EGFR localization at focal adhesions, indicating that uPAR regulates RTK membrane organization rather than only kinase activity.^64^ Mechanistically, uPAR-induced integrin clustering and Src activation drive EGFR phosphorylation at Y845 (a Src-specific site), triggering EGF-independent signaling.

In VSMCs, uPAR engagement induces PDGFR-β phosphorylation and ERK (extracellular signal-regulated kinase)-dependent proliferation, directly relevant to neointimal hyperplasia and adverse cardiac remodeling.^52,65,66^ RTK transactivation may represent a mechanism by which uPAR amplifies mitogenic signals in the vascular wall without requiring growth factor ligand, a potentially important distinction in growth-factor-depleted microenvironments of advanced atherosclerotic plaques.

### RAGE and FPRs: Emerging Coreceptors in the Membrane Context

Two additional coreceptor families, characterized in soluble suPAR signaling (see section suPAR as an Endocrine-Like Mediator of Inflammation), may also partner with membrane uPAR. RAGE physically associates with αvβ3 in podocytes,^14^ and both uPAR and RAGE are raft-enriched on endothelial, myeloid, and podocytes.^3,67,68^ Whether membrane-tethered uPAR engages RAGE in cis has not been tested, but if confirmed would imply that the local-to-diffuse transition (see section Cleavage, Release, and Generation of suPAR Isoforms) changes geometry and reach of signaling rather than the coreceptor requirement. FPR1 also coimmunoprecipitates with β3-integrin subunits in podocytes,^23^ and the D2D3 chemotactic epitope is present on intact membrane uPAR before cleavage, raising the possibility of cis-engagement. Which coreceptor partnerships are exclusive to membrane uPAR, exclusive to suPAR, or shared with different signaling consequences remains a priority (Table 1).

**Table 1.**
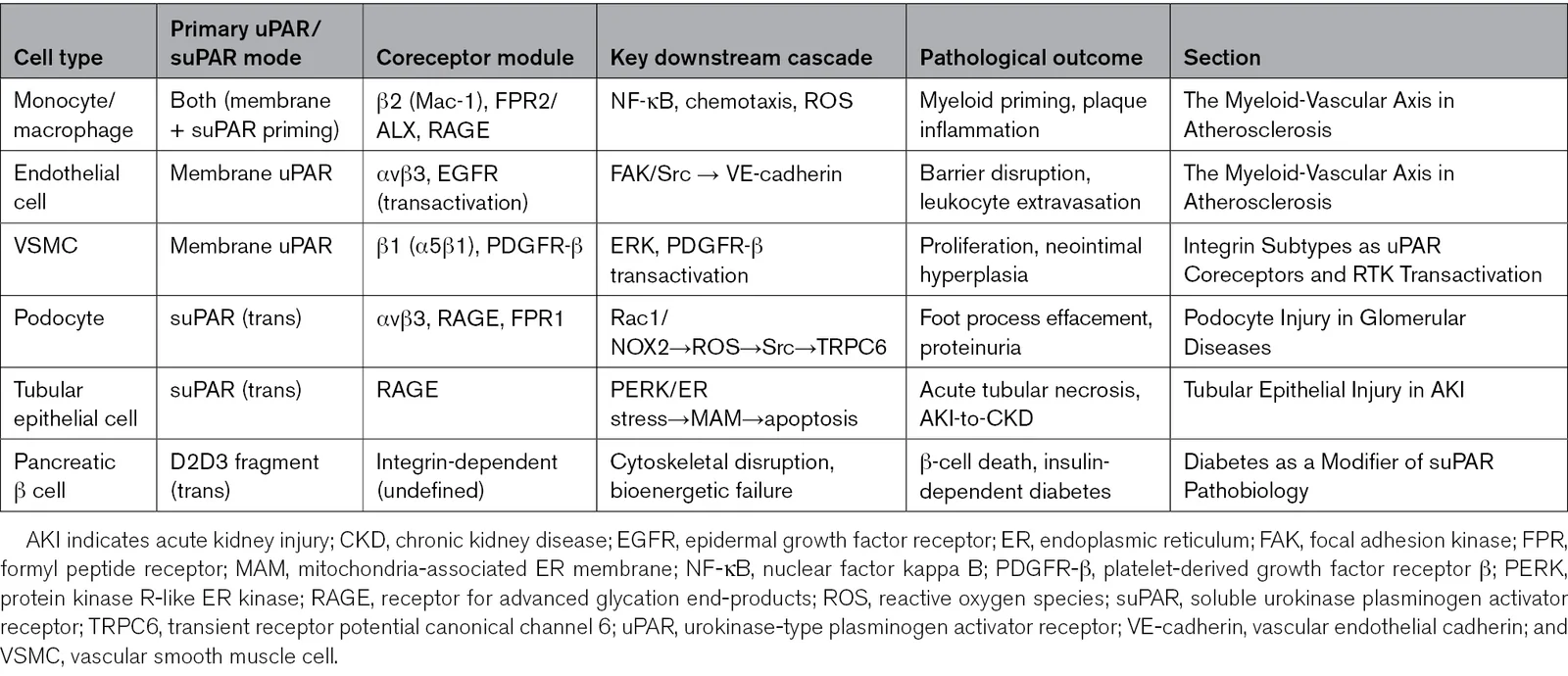
Coreceptor Partnerships and Signaling Outcomes by Cell Type

## suPAR as an Endocrine-Like Mediator of Inflammation

### Integrins as Cell Surface Receptors for suPAR

Once released from the cell surface (see section Cleavage, Release, and Generation of suPAR Isoforms), suPAR retains the capacity to engage integrins on distant target cells, converting a local membrane scaffold into a circulating agonist with endocrine-like reach. Surface plasmon resonance demonstrates that suPAR forms a high-affinity complex with αvβ3-integrin,^49^ and functional studies confirm that this interaction activates integrin-dependent signaling in multiple cell types.^14,69–72^ Pharmacological blockade of αvβ3-integrin with cilengitide or endogenous antagonists, such as ICOSL (inducible costimulator ligand), prevents suPAR-evoked cellular injury^72,73^ in patients with recurrent primary focal segmental glomerulosclerosis (FSGS), therapies that reduce suPAR also reduce glomerular β3-integrin activation, and the degree of de-activation FSGS correlates with treatment response.^70,74^

A critical distinction between membrane uPAR and suPAR integrin engagement merits emphasis. Membrane uPAR activates integrins laterally, in cis, regulated by local coreceptor availability, lipid raft dynamics, and conformational state (see section uPAR as a Membrane Signaling Scaffold). suPAR activates integrins in trans, on cells that may be anatomically remote from the site of shedding. The shift from cis to trans engagement removes the spatial constraints that normally limit uPAR signaling and exposes cell types that may not express membrane uPAR to integrin activation, helping to explain the multiorgan reach of CKM syndrome.

### RAGE as a suPAR Coreceptor

**Figure 4.**
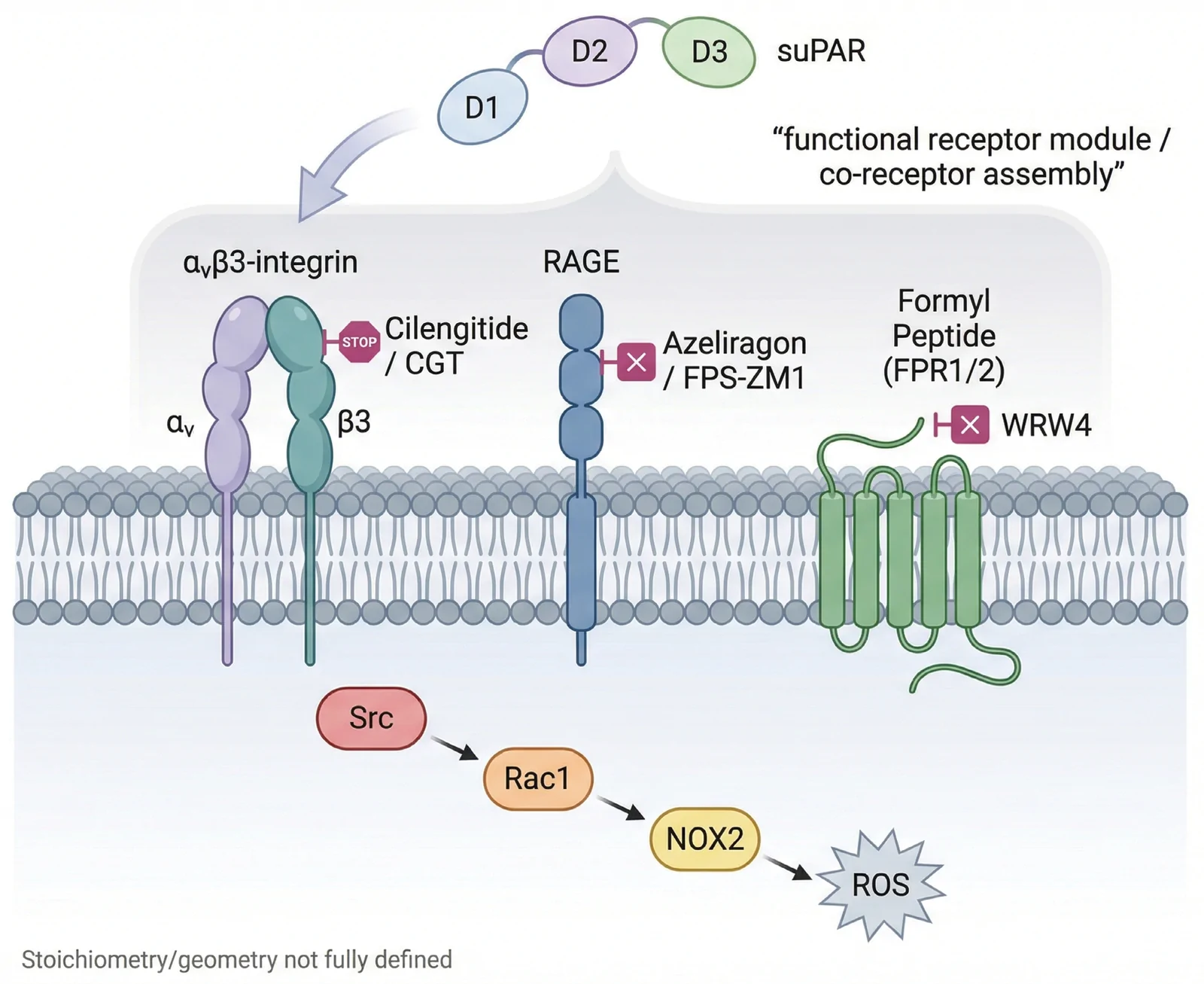
**suPAR (soluble urokinase plasminogen activator receptor)-responsive coreceptor module on target cells.** Schematic of a suPAR-responsive surface module involving αvβ3-integrin, receptor for advanced glycation end-products (RAGE), and FPRs (formyl peptide receptors) with representative downstream signaling nodes and experimentally used antagonists. FPS-ZM1 indicates high-affinity small-molecule RAGE antagonist; NOX2, NADPH oxidase 2; Rac1, Ras-related C3 botulinum toxin substrate 1; ROS, reactive oxygen species; and WRW4, Trp-Arg-Trp-Trp (FPR antagonist).

The RAGE functions as an essential coreceptor for suPAR signaling. Coimmunoprecipitation studies demonstrate a direct interaction between endogenously expressed RAGE and αv and β3 integrin subunits in podocytes, and siRNA knockdown of RAGE completely abolishes suPAR-evoked activation of the Rac1–Src–TRPC6 (transient receptor potential canonical channel 6) signaling cascade^14^ (Figure 4). Selective small-molecule RAGE inhibitors (azeliragon, FPS-ZM1 [high-affinity small-molecule RAGE inhibitor]) also block suPAR signaling and are as effective as Arg-Gly Asp integrin inhibitors, such as cilengitide.^14,71,72^ These data indicate that αvβ3-integrin and RAGE function as obligate coreceptors for suPAR.

RAGE engagement differs from the canonical AGE (advanced glycation end-product)–RAGE axis in 3 ways: (1) suPAR requires αvβ3-integrin as an obligate coreceptor, whereas AGEs activate RAGE through direct binding; (2) suPAR-RAGE activates Src and Rac1 leading to TRPC6 mobilization, a branch absent from AGE–RAGE signaling but central to podocyte injury; and (3) circulating suPAR reflects immune activation, whereas AGEs accumulate with glycemic burden. The 2 share downstream effectors but operate on different time scales and disease contexts.

The RAGE coreceptor relationship extends beyond podocytes. In HK-2 (human kidney 2) TEC, suPAR engages RAGE to promote endoplasmic reticulum (ER) stress, mitochondrial dysfunction, and apoptosis, all key pathological features of sepsis-induced acute kidney injury (AKI).^22^ Whether suPAR–RAGE signaling operates similarly in endothelial cells and macrophages, cell types central to atherosclerosis, has not been tested but would substantially broaden the relevance of this coreceptor axis to CKM pathophysiology.

### Formyl Peptide Receptors in suPAR Signaling

#### Myeloid Chemotaxis Through FPR2/ALX

FPRs (formyl peptide receptors) are a third coreceptor class contributing to suPAR signaling. In myeloid cells, D2D3 binds FPR2/ALX and triggers Gαi-mediated signaling leading to Ca2^+^ mobilization, actin polymerization, and directed chemotaxis.^8,75^ This provides a molecular explanation for urokinase-stimulated cell migration independent of its proteolytic activity. In CKM syndrome, FPR-mediated chemotaxis driven by D2D3 could contribute to monocyte recruitment to atherosclerotic plaques and neutrophil infiltration in AKI^8^ (Figure 4).

An intriguing feature of FPR2/ALX is its capacity for biased agonism: peptide agonists, including D2D3 drive proinflammatory responses, whereas specialized proresolving lipid mediators (lipoxin A4, resolvin D1) drive anti-inflammatory responses through the same receptor.^75,76^ Whether elevated D2D3 in chronic CKM conditions competes with proresolving ligands for FPR2/ALX occupancy, thereby sustaining inflammation by impeding resolution, represents a testable hypothesis. Targeting FPR1/2 with selective antagonists or biased agonists may offer a strategy to modulate leukocyte trafficking with greater specificity than broad anti-inflammatory approaches.^77,78^

#### Time-Dependent Biased Signaling Through FPRs in Podocytes

FPR1 is expressed in mouse podocytes, where it coimmunoprecipitates with endogenously expressed β3-integrin subunits, indicating that FPRs form part of the multireceptor suPAR-responsive complex alongside αvβ3-integrin and RAGE^23^ (Figure 4). Pharmacological dissection reveals time-dependent biased signaling. Classical FPR agonists such as N-formylmethionyl-leucyl-phenylalanine evoke reactive oxygen species (ROS) and Src activation at both 60-minute and 24-hour exposures, both blocked by the FPR antagonist WRW4 (Trp-Arg-Trp-Trp). The early response is pertussis toxin-sensitive (canonical Gαi), whereas the late response is pertussis toxin-resistant but abolished by β-arrestin-1 knockdown.^23^ This switch from G-protein- to β-arrestin-dependent coupling resembles biased GPCR kinetics described elsewhere, but its demonstration in a multi-receptor complex involving integrins is novel.

suPAR also activates podocyte FPRs, but only at the 24-hour time point, with responses that are pertussis toxin-resistant and β-arrestin-1-dependent.^23^ One interpretation is that sustained suPAR exposure drives β-arrestin-1-dependent FPR internalization, leading to transactivation of αvβ3-integrin and possibly RAGE. The finding that suPAR signals through FPRs via a β-arrestin-dependent, G protein-independent pathway raises the possibility that FPR-targeted biased agonists could selectively modulate specific branches of suPAR signaling. One caveat: mouse FPR1 (≈77% homology with human FPR1) shares structural and functional features with human FPR2/ALX,^79^ so validation in human FPR biology is required.

## Intracellular Signaling Modules of uPAR and suPAR

Coreceptor assemblies (see sections uPAR as a Membrane Signaling Scaffold and suPAR as an Endocrine-Like Mediator of Inflammation) converge on shared effectors (Src-family kinases, Rac1, NF-κB [nuclear factor kappa B]), but the engagement mode determines which predominates. Membrane uPAR favors FAK/RTK-driven migration and proliferation (see sections Src/FAK Signaling and Downstream Kinase Cascades and Rho GTPase Regulation and Cytoskeletal Plasticity); soluble suPAR activates a NOX2 (NADPH oxidase 2)-ROS-Src-TRPC6 cascade with distinct pathology (see section suPAR-Activated Signaling Cascades); NF-κB-driven *PLAUR* feedback amplifies both (see section Transcriptional Amplification of *PLAUR* Expression).

### Src/FAK Signaling and Downstream Kinase Cascades

Intracellular signaling downstream of membrane uPAR is initiated by Src-family kinases, particularly Fyn and Src, enriched in lipid raft microdomains.^3,5^ Urokinase binding triggers rapid FAK phosphorylation at Y397, creating a docking site for Src and forming an FAK-Src complex that phosphorylates focal adhesion substrates, including paxillin, p130Cas, and cortactin.^5^ The resulting uPAR → FAK → Src axis controls migration via focal adhesion turnover. The uPA ATF (amino-terminal fragment) is sufficient to induce FAK/Src activation, establishing a receptor-mediated, protease-independent mechanism.^5,80^

uPAR engagement also activates canonical kinase cascades regulating proliferation, survival, and metabolism. MAPK (mitogen-activated protein kinase)/ERK signaling is activated through both Ras-dependent and Ras-independent mechanisms,^5,52^ while PI3K (phosphoinositide 3-kinase)/Akt provides parallel survival signals downstream of uPAR-integrin coupling.^81,82^ uPAR-mediated RTK transactivation (see section RTK Transactivation) drives ligand-independent EGFR and PDGFR-β signaling: Src-mediated EGFR phosphorylation at Y845 triggers downstream EGFR cascades, while PDGFR-β transactivation drives ERK-dependent VSMC proliferation relevant to vascular remodeling.^52,61,65^ The CKM relevance: ERK activation drives VSMC proliferation and dedifferentiation in neointimal hyperplasia, while PI3K/Akt-dependent metabolic reprogramming may support the inflammatory macrophage phenotype in atherosclerotic plaque.

### Rho GTPase Regulation and Cytoskeletal Plasticity

uPAR controls cell morphology, migration, and barrier function through Rho-family GTPases.^66,83^ Vitronectin binding activates a p130Cas → CrkII → DOCK180 → Rac1 axis promoting actin polymerization at the leading edge; dominant-negative Rac1 blocks uPAR-induced lamellipodia.^30,84,85^ A functionally important feature is context-dependent plasticity: uPAR promotes RhoA inactivation and Rac1-dependent protrusion during mesenchymal migration but favors RhoA activation and actomyosin contractility during ameboid migration.^86^ Such plasticity allows migration mode switching in response to microenvironmental cues, with direct relevance to macrophage navigation between matrix-dense fibrous caps and necrotic cores in atherosclerotic plaques, and to podocyte foot process dynamics whose dysregulation underlies effacement. Rac1 is also a key effector of suPAR signaling in podocytes (see section suPAR-Activated Signaling Cascades), where it activates NOX2 and drives ROS generation; the same GTPase thus mediates cytoskeletal protrusion in migrating cells but oxidative stress in podocytes, depending on context.

### Transcriptional Amplification of *PLAUR* Expression

Activation of NF-κB and JAK (Janus kinase)-STAT (signal transducer and activator of transcription) pathways downstream of uPAR-integrin-RAGE signaling upregulates the *PLAUR* gene encoding uPAR, creating a positive feedback loop in which receptor activation increases receptor expression.^7,8,87–89^ This feed-forward mechanism has been demonstrated in cancer cells but not yet in CKM-relevant cell types, though it is plausible given shared signaling machinery in macrophages and podocytes, where suPAR–RAGE signaling activates NF-κB.^14^

Clinical observations support a feed-forward model: CKD patients show progressively rising suPAR that tracks disease progression.^1,10^ Circulating concentrations are typically 2 to 3 ng/mL in healthy individuals versus 5 to 15 ng/mL or higher in advanced CKD, sepsis, or active FSGS,^1,13,17^ overlapping the 1 to 10 ng/mL range used in cell-based studies.^49,69,72^ suPAR is not alone in engaging these effectors (TNF-α, AGEs also converge on NF-κB, Src, Rac1), but its distinctive contribution is direct engagement of the αvβ3-integrin–RAGE module,^71^ providing a specific molecular bridge between systemic immune activation and end-organ integrin signaling. Regional plasma sampling shows only modest renal suPAR extraction,^90^ implicating production rather than impaired clearance as the dominant source of rising levels.

### suPAR-Activated Signaling Cascades

Although the transcriptional programs above amplify receptor availability, the acute signaling events triggered by suPAR engagement of its coreceptor module are equally important and have been most extensively mapped in podocytes.^17,49^ suPAR engagement of the αvβ3-integrin–RAGE coreceptor module initiates a well-characterized sequence: Rac1 activation drives NOX2 assembly and cytosolic ROS generation.^72^ Aberrant Rac1 activation in podocytes contributes to foot process effacement and proteinuria.^91^ ROS in turn activates Src, an effect reduced by pretreatment with 4-hydroxy-2,2,6,6-tetramethylpiperidine-1-oxyl and mimicked by exogenous H_2_O_2_.^72^ Src activation then drives mobilization of Ca2^+^-permeable TRPC6 channels to the cell surface, a step blocked by the Src inhibitor PP1.^72^ Consistent with a causal role for this cascade in vivo, Src inhibitors reduce proteinuria in mice overexpressing suPAR variants.^91–93^

TRPC6-mediated Ca2^+^ influx is a critical amplification step. Elevated intracellular Ca2^+^ activates calcineurin-nuclear factor of activated T cells cascades within the cell body, promotes cytoskeletal reorganization in foot processes, and can trigger apoptotic cell death.^91^ Both D2D3 and full-length suPAR mobilize TRPC6.^72^ suPAR signaling also reduces the abundance of essential slit diaphragm proteins, including podocin, whose loss increases podocyte mechanosensitivity and further enhances TRPC6 activation, creating a feed-forward loop that amplifies injury.^72^ suPAR can additionally activate metalloproteases, leading to shedding of nephrin ectodomains,^94^ extending consequences to direct degradation of filtration barrier structural proteins. ROS likely activates MMPs by oxidizing the propeptide cysteine switch that maintains latency; disruption of cysteine-zinc coordination exposes the catalytic site and converts pro-MMPs to active forms.^95^ The same mechanism operates in macrophage-derived foam cells, where ROS and reactive nitrogen species activate MMP-2 and MMP-9 to thin the fibrous cap and destabilize plaque.^96^ The suPAR → NOX2 → ROS → MMP axis may therefore represent a conserved module operating in the kidney (nephrin shedding, barrier degradation) and the vasculature (matrix degradation, plaque rupture).

The complete podocyte cascade (Rac1/NOX2 → ROS → Src → TRPC6 → Ca2^+^ → calcineurin/nuclear factor of activated T cells, with parallel slit diaphragm protein loss) identifies multiple pharmacologically tractable nodes. Whether this cascade operates in other αvβ3-expressing cell types is an open question, though suPAR-induced Src/ROS in tubular cells via RAGE (see section RAGE as a suPAR Coreceptor) and broad TRPC6 expression suggest partial conservation.

## Cell-Type and Tissue Programs in CKM Syndrome

**Figure 5.**
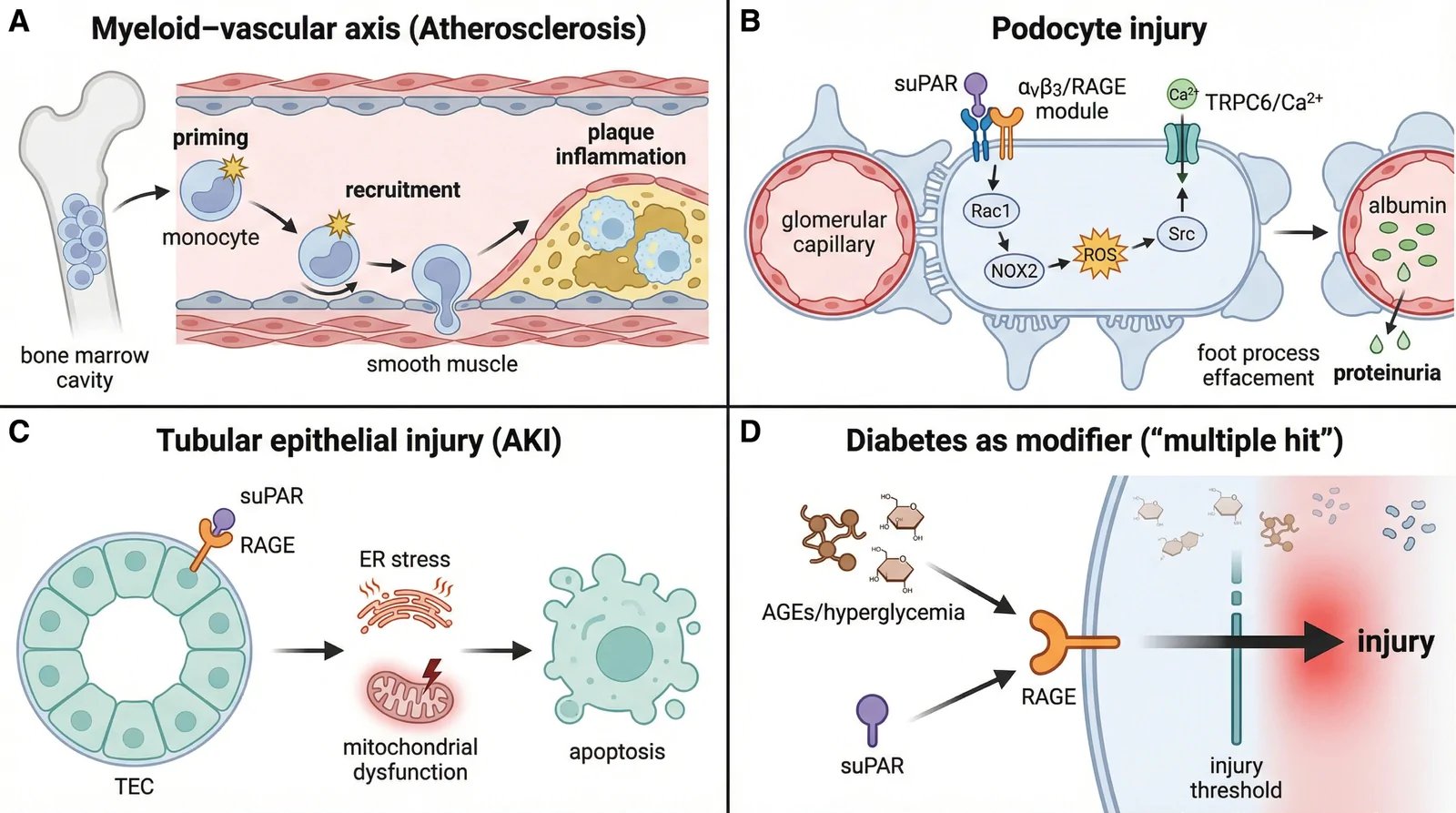
**Cell-type programs linked to suPAR (soluble urokinase plasminogen activator receptor) in cardiovascular-kidney-metabolic (CKM) syndrome. A**, Myeloid–vascular axis in atherosclerosis. **B**, Podocyte injury. **C**, Tubular epithelial injury in acute kidney injury (AKI). **D**, Diabetes as a modifier via convergent receptor for advanced glycation end-products (RAGE) signaling. AGE indicates advanced glycation end-product; AKI, acute kidney injury; ER, endoplasmic reticulum; ROS, reactive oxygen species; TEC, tubular epithelial cell; and TRPC6, transient receptor potential canonical channel 6.

The signaling machinery described in sections uPAR as a Membrane Signaling Scaffold to Intracellular Signaling Modules of uPAR and suPAR is largely shared across cell types (Table 1), yet the pathological outcomes are different: myeloid priming and plaque formation in the vasculature, foot process effacement in the glomerulus, ER stress and apoptosis in tubular epithelium, and amplified multi-organ injury in diabetes. The divergence arises because the same molecular signals interact with cell-type-specific architecture to produce distinct disease phenotypes (Figure 5).

What determines which coreceptor module and downstream branch predominates in each cell type? Four nonexclusive mechanisms contribute. (1) Differential coreceptor expression establishes distinct signaling-competent surfaces: podocytes are αvβ3-high,^97,98^ myeloid cells are enriched in β2 integrins (Mac-1), and FPR2/ALX, and endothelial cells express both αvβ3 and EGFR available for transactivation. (2) Cell-type-specific scaffolds organize signaling complexes within distinct membrane subdomains: in podocytes, podocin tethers TRPC6 channels to NOX2 within the slit diaphragm, a molecular architecture absent from tubule and myeloid cells^99^; β-arrestin-1, which mediates biased FPR signaling in podocytes (see section Formyl Peptide Receptors in suPAR Signaling),^23^ may selectively couple FPRs to integrin transactivation rather than canonical G protein pathways, with a role that varies across cell types. (3) Mechanical environment differs substantially: podocytes experience hemodynamic filtration forces that lower thresholds for integrin-mediated cytoskeletal disruption,^100^ endothelial cells experience shear stress that activates mechanosensitive FAK signaling, and myeloid cells navigate variable matrix stiffness during transmigration. (Iv) Temporal dynamics matter: the same initial signal may produce cytoskeletal remodeling over minutes (myeloid migration) versus cumulative injury over months (podocyte effacement), depending on regenerative capacity and exposure duration. This question remains one of the most important open problems in uPAR biology; systematic single-cell spatial transcriptomics of coreceptor modules across CKM-relevant tissues could resolve it.

### The Myeloid-Vascular Axis in Atherosclerosis

#### Myeloid Priming by suPAR

The uPAR/suPAR axis participates in multiple stages of atherogenesis, beginning with myeloid priming, during which multiple myeloid cell populations are affected. Classical monocytes (CD14++CD16−) and intermediate monocytes (CD14++CD16+) are the principal circulating responders: uPAR on these cells facilitates firm adhesion to inflamed endothelium through Mac-1 (αMβ2/CD11b-CD18) engagement, while suPAR and its D2D3 fragment drive directed chemotaxis through FPR2/ALX-mediated Gαi signaling, triggering Ca2^+^ mobilization and actin polymerization.^8,53,75^ Neutrophils contribute indirectly by shedding the chemotactically active D2D3 fragment on activation, which in turn recruits and activates monocytes via FPR1/2. Within the vessel wall, plaque macrophages of the inflammatory phenotype express high levels of uPAR, which promotes matrix migration via Rac1-dependent cytoskeletal remodeling (see section Rho GTPase Regulation and Cytoskeletal Plasticity), enhances oxidized LDL uptake and foam cell formation, and drives secretion of IL-6, IL-1β, TNF-α, and MMPs that expand plaque volume and thin the fibrous cap.^11,88,96^ Importantly, ROS generated by macrophage NOX2 can activate MMP-2 and MMP-9 through the cysteine switch mechanism (see section suPAR-Activated Signaling Cascades),^95^ directly linking uPAR-dependent ROS production to plaque destabilization.

Individuals with elevated suPAR have worse cardiovascular outcomes, greater coronary artery calcification, and monocytes with enhanced chemotactic responses.^11^ In transgenic mice overexpressing suPAR, aortic monocyte counts and CCL2 (C-C motif chemokine ligand 2) secretion are elevated before atherosclerosis induction. Following PCSK9 (proprotein convertase subtilisin/kexin type 9)-mediated hypercholesterolemia, transgenic mice overexpressing suPAR develop larger plaques with necrotic cores and extensive macrophage infiltration compared with controls, despite equivalent cholesterol levels. The mechanistic basis involves at least 3 converging pathways: (1) Mac-1 activation promoting firm endothelial adhesion and transendothelial migration^53,101^; (2) RAGE-mediated NF-κB activation^11,101,102^ driving cytokine amplification and sustained inflammatory gene expression^14,102^; and (3) FPR2/ALX engagement by D2D3 directing chemotaxis toward sites of plaque inflammation.^8,75^ This phenotype resembles trained immunity, but whether suPAR directly induces the epigenetic reprogramming (H3K4me3, H3K27ac at inflammatory loci) characteristic of trained immunity remains untested.^103,104^

#### Endothelial Barrier Disruption

suPAR-primed monocytes encounter an endothelium that is itself rendered permeable by uPAR signaling. uPAR regulates vascular endothelial cadherin-based adherens junctions, controlling barrier permeability.^105^ Inflammatory cytokines upregulate endothelial uPAR expression and promote vascular endothelial cadherin phosphorylation and endocytosis, leading to junction opening and enhanced leukocyte extravasation.^106–108^ The mechanism likely involves the same FAK/Src pathway operative in other uPAR-responsive cell types (see section Src/FAK Signaling and Downstream Kinase Cascades): uPAR-dependent FAK activation in endothelial cells phosphorylates junctional substrates that destabilize vascular endothelial cadherin complexes, amplifying cytokine-induced barrier disruption. suPAR may further compromise endothelial integrity through direct activation of endothelial αvβ3-integrin or RAGE, though this trans-acting mechanism requires additional validation. The convergence of suPAR-primed monocytes and a uPAR-weakened endothelial barrier creates a permissive environment for leukocyte extravasation, the critical initiating event in atherogenesis.

#### Subendothelial Macrophage Biology and Plaque Progression

Once within the vessel wall, macrophages express membrane uPAR that enhances matrix migration through Rho GTPase pathways (see section Rho GTPase Regulation and Cytoskeletal Plasticity) and promotes oxidized lipoprotein uptake.^11,88^ Deficiency of uPAR in hematopoietic cells reduces atherosclerosis in mouse models, confirming a causal role for myeloid uPAR in atherogenesis.^109^ The uPAR/suPAR axis thus contributes at every stage of atherogenesis, from circulating monocyte priming through endothelial transmigration to subendothelial macrophage accumulation, providing several intervention points for a single molecular target (see section Therapeutic Implications).

#### Cross-Cell-Type Applicability of the Podocyte Cascade

The podocyte Rac1/NOX2 → ROS → Src → TRPC6 cascade (see section suPAR-Activated Signaling Cascades) is likely not podocyte-specific. NOX2 dominates phagocytic ROS generation; TRPC6 contributes to macrophage phagosomal function, leukocyte chemotaxis and cytokine release,^110^ and chemokine-directed neutrophil migration^111^; and TRPC6 upregulation cooccurs with ER stress in glucolipotoxic monocytes.^112^ These observations raise the possibility that suPAR-driven NOX2-ROS-TRPC6 activation in myeloid cells amplifies inflammation within vulnerable atherosclerotic plaques, while ER stress (the hallmark of tubular suPAR signaling in section Tubular Epithelial Injury in Acute Kidney Injury) may be a general consequence of sustained suPAR engagement rather than a tubule-specific effect. Whether the full podocyte cascade is conserved or branches differently across cell types is a critical open question for target selection (see section Downstream Pathway Targeting).

### Podocyte Injury in Glomerular Diseases

#### Why Podocytes Are Uniquely Vulnerable

Podocytes are terminally differentiated epithelial cells whose interdigitating foot processes form the slit diaphragm, the most selective component of the glomerular filtration^113,114^ barrier. Several features render podocytes uniquely susceptible to suPAR-induced injury^49,70,100,115^: their postmitotic nature severely limits regenerative capacity; they express high levels of αvβ3-integrin relative to other kidney^97,98^ cells; they are subjected to considerable hemodynamic forces creating a low threshold for integrin-mediated cytoskeletal^100,116^disruption; and suPAR injury is potentiated by additional pathogenic factors including *APOL1* (apolipoprotein L1) risk variants and coexposure to TNF, suggesting that suPAR operates as 1 component of a multihit model in many glomerular diseases.^71,72,74^

#### From suPAR Signaling to Filtration Barrier Disruption

Binding of suPAR to the αvβ3-integrin–RAGE–FPR coreceptor module (see sections Integrins as Cell Surface Receptors for suPAR and Formyl Peptide Receptors in suPAR Signaling) activates the Rac1/NOX2 → ROS → Src → TRPC6 cascade (see section suPAR-Activated Signaling Cascades). TRPC6-mediated Ca2^+^ influx activates calcineurin-nuclear factor of activated T cells cascades and promotes cytoskeletal reorganization, resulting in foot process effacement.^91^ In parallel, suPAR signaling reduces podocin and nephrin abundance.^18,72,94^ Podocin loss increases podocyte mechanosensitivity and further enhances TRPC6 activation, while TRPC6 activates metalloproteases that shed nephrin ectodomains.^94^ The combined result is foot process effacement, slit diaphragm disassembly, and podocyte apoptosis, producing the severe proteinuria characteristic of nephrotic syndrome.

#### Clinical Evidence in FSGS

Primary FSGS patients frequently exhibit elevated plasma suPAR levels, and elevated suPAR predicts disease recurrence following kidney transplantation.^70,117^ Exogenous suPAR induces FSGS-like glomerulopathy in mice, and β3-integrin knockout confers protection.^70^ Lowering suPAR by plasmapheresis or αvβ3 blockade attenuates injury,^70,117^ and reductions in suPAR-mediated β3-integrin activation correlate with treatment response.^74^ Collectively, these data support suPAR as a pathogenic driver in a subset of FSGS phenotypes, though it is not a universal explanation for all forms of the disease.

### Tubular Epithelial Injury in AKI

Although podocytes are the primary target in chronic glomerular disease, TECs bear the brunt of suPAR-mediated injury in AKI, particularly sepsis-associated AKI.^19,118^ Sepsis-associated AKI affects approximately two-thirds of critically ill patients and carries high mortality with frequent progression to CKD. In polymicrobial sepsis models, suPAR levels rise rapidly and correlate with kidney injury severity, and genetic deletion or antibody-mediated neutralization of suPAR ameliorates sepsis-associated AKI.^19^

The intracellular pathway in TECs diverges from that of podocytes. Whereas suPAR activates Rac1/NOX2 → ROS → Src → TRPC6 in podocytes, in TECs, suPAR engages RAGE to trigger ER stress and mitochondrial dysfunction, culminating in caspase-3-mediated apoptosis.^22^ The ER stress arm involves the PERK (protein kinase R-like ER kinase)–eIF2α–activating transcription factor 4/CHOP axis, which propagates to mitochondria via disrupted mitochondria-associated ER membrane contacts, resulting in ROS production, membrane potential collapse, and cytochrome c release.^119–122^ Both cascades require RAGE and generate ROS, but the upstream effectors and end points are distinct, illustrating how the same receptor module produces cell-type-specific pathology.

AKI through this pathway can transition to CKD. Surviving TECs adopt a senescent, proinflammatory phenotype characterized by cell-cycle arrest and a senescence-associated secretory phenotype that perpetuates inflammation and fibrosis.^123,124^ uPAR has been identified as a broadly upregulated cell-surface marker of senescence, with uPAR-positive senescent cells accumulating across multiple tissues during aging and fibrosis.^125,126^ Whether suPAR sustains maladaptive repair through RAGE–NF-κB signaling engaging the *PLAUR* feed-forward loop, and whether targeted elimination of uPAR-positive senescent TECs could interrupt the AKI-to-CKD transition, represents a testable therapeutic hypothesis (see section Senolytic Immunotherapy Targeting uPAR-Positive Cells).

### Diabetes as a Modifier of suPAR Pathobiology

#### The Second Hit Model

Diabetes accelerates atherosclerosis, increases heart failure risk, and is the leading cause of CKD.^127^ Traditional mechanisms fail to explain why some diabetic patients develop severe complications while others remain protected despite similar glycemic control, implying that organ damage often requires a second hit that amplifies the diabetic milieu beyond a threshold for irreversible injury. We propose that suPAR functions as such a synergistic second hit and may simultaneously worsen diabetes itself, creating a bidirectional vicious cycle.

The diabetic milieu primes the cell: hyperglycemia-generated AGEs tonically activate RAGE, producing baseline NF-κB, ROS, and endothelial dysfunction, while diabetic inflammation enhances *PLAUR* transcription and increases uPAR expression and suPAR shedding,^7^ with adipose and hepatic macrophages serving as likely reservoirs.^130,131^ Superimposed suPAR may create a combinatorial signal at RAGE that exceeds thresholds for irreversible injury.^128,129^ Epidemiologically, suPAR predicts cardiovascular and kidney outcomes in diabetes independently of hemoglobin A1c, blood pressure, and lipids^1,130,131^; diabetic mice show enhanced susceptibility to suPAR-induced kidney injury,^13,132^ and TNF-α and suPAR effects on podocytes are additive or greater.^71^

#### suPAR as a Cause of Diabetes: the D2D3-β Cell Axis

The relationship between suPAR and diabetes is not unidirectional: D2D3 directly injures pancreatic β cells.^12^ High-fat-fed transgenic mice expressing D2D3 develop insulin-dependent diabetes with decreased insulin and C-peptide, impaired glucose-stimulated insulin secretion, reduced β-cell mass, and elevated fasting glucose. At the cellular level, D2D3 impairs β cell proliferation, disrupts bioenergetics, dysregulates cytoskeletal dynamics, and blunts insulin granule maturation and trafficking, linking suPAR-driven cytoskeletal disruption to the integrin-dependent pathways active in podocytes and vascular cells. Anti-uPAR antibody restored β-cell mass and function, establishing reversible suPAR-dependence. D2D3 predominates in sera from patients with nephropathy and insulin-dependent diabetes, and these sera inhibit glucose-stimulated insulin release from human islets in a D2D3-dependent manner.^12^

These findings transform the second hit model into a bidirectional vicious cycle: diabetes-associated inflammation increases suPAR shedding, while D2D3 destroys β cells and worsens glycemic control. This cycle may explain why suPAR predicts diabetic complications independently of glycemic control.

The diabetic kidney also illustrates the coreceptor selectivity principle introduced in section Domain Architecture of uPAR and Its Conformational Dynamics in a disease-relevant context. FXII (factor XII), elevated in diabetic plasma, has been identified as a uPAR ligand that induces integrin β1-dependent (rather than αvβ3-dependent) signaling in TEC, driving oxidative stress, DNA damage, and cellular senescence.^24^ The coexistence of suPAR–αvβ3 and FXII–uPAR–β1 signaling in the same tissue demonstrates that different uPAR ligands can select different integrin partners and produce different pathological end points in vivo, providing a concrete example of the biased signaling model operating through coreceptor selection. From a therapeutic standpoint, this ligand-integrin multiplicity implies that targeting a single integrin subtype may be insufficient to fully interrupt uPAR-mediated injury in diabetes.

## Therapeutic Implications

The distinction between local and systemic signaling modes guides therapeutic target selection. Disrupting the receptor interface (see section Targeting the Receptor-Ligand Interface) can block both modes at their origin. Neutralizing suPAR (see section SuPAR Removal and Neutralization) selectively intercepts the soluble arm. Eliminating uPAR-positive senescent cells (see section Senolytic Immunotherapy Targeting uPAR-Positive Cells) addresses the cellular source of suPAR production. Targeting downstream effectors (see section Downstream Pathway Targeting) interrupts shared intracellular cascades with progressively broader on-target effects (Table 2).

**Table 2.**
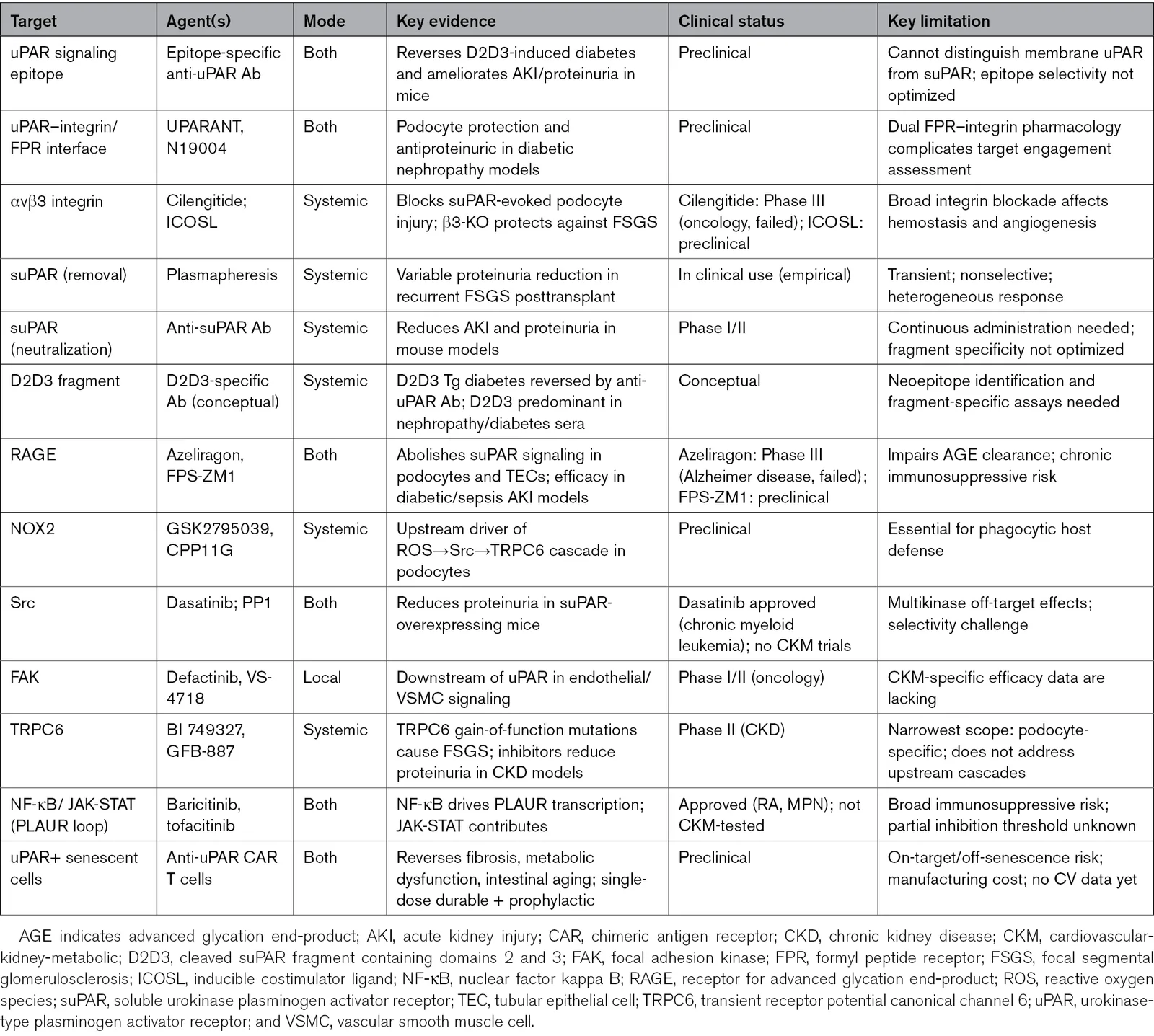
Pharmacologically Tractable Nodes in the uPAR/suPAR Signaling Axis

### Targeting the Receptor-Ligand Interface

#### Epitope-Specific Antibodies

Structure-function studies (see section Domain Architecture of uPAR and Its Conformational Dynamics) have mapped discrete functional epitopes: the central cavity accommodates urokinase, D3, and the D1-D2 linker mediate integrin interactions, and the vitronectin-binding surface overlaps with D3.^133,134^ Antibodies targeting the integrin-binding interface, including the GEEG motif (see section Domain Architecture of uPAR and Its Conformational Dynamics), might selectively inhibit pathological signaling while preserving urokinase–uPAR interaction and plasminogen activation. Because suPAR is biochemically identical to uPAR minus its glycosylphosphatidylinositol anchor, selectivity between the 2 signaling modes depends on pharmacokinetic properties rather than epitope specificity: a systemically administered antibody would primarily neutralize suPAR while also engaging membrane uPAR on vascular-accessible cells. A potential exception involves D2D3-specific antibodies: if neoepitopes are exposed at the D1 cleavage site, fragment-selective antibodies could preferentially target the D2D3 species implicated in β-cell destruction and nephropathy (see section Diabetes as a Modifier of suPAR Pathobiology).

Preclinical studies have demonstrated efficacy in FSGS and AKI models.^13,70^ The most striking proof-of-concept comes from the D2D3 transgenic model, in which anti-uPAR antibody reversed insulin-dependent diabetes,^12^ demonstrating that suPAR-driven organ injury can be reversed by antibody-mediated targeting even in metabolic organs not traditionally associated with the uPAR axis.

#### Small-Molecule Receptor Interface Inhibitors

UPARANT and N19004, inhibitors of the uPAR–integrin interaction, preserve podocyte structure and reduce proteinuria in preclinical models.^135,136^ Both compounds also function as effective FPR antagonists,^137–139^ consistent with the coimmunoprecipitation of FPR1 with β3-integrin subunits in podocytes (see section Formyl Peptide Receptors in suPAR Signaling). This dual FPR–integrin pharmacology could be advantageous, providing more complete blockade of suPAR signaling than targeting either receptor alone.

### suPAR Removal and Neutralization

Direct removal of suPAR by plasmapheresis has been applied empirically in recurrent FSGS posttransplantation, with variable results likely reflecting etiologic differences; only suPAR-mediated FSGS should respond.^117^ A more fundamental limitation is pharmacokinetic: suPAR is continuously produced (see section Transcriptional Amplification of *PLAUR* Expression), making reductions by plasmapheresis inherently transient. Plasmapheresis is, therefore, best understood as a bridge therapy while longer-acting interventions take effect.

Neutralizing antibodies that prevent suPAR interaction with integrins and RAGE could achieve more sustained suppression. Proof-of-concept studies demonstrate efficacy in AKI and proteinuria models.^13,14,22^ An important design consideration is fragment specificity: since D2D3 has distinct pathogenic activities, including β-cell destruction (see section Diabetes as a Modifier of suPAR Pathobiology) and is the predominant species in patients with nephropathy and insulin-dependent diabetes,^12^ fragment-specific antibodies targeting D2D3 could offer more precise intervention. Clinical translation will require defined epitope selectivity, acceptable immunogenicity, and fragment-specific target-engagement biomarkers.

### Senolytic Immunotherapy Targeting uPAR-Positive Cells

An orthogonal approach exploits uPAR’s upregulation in senescent cells. Cellular senescence, characterized by stable cell-cycle arrest and senescence-associated secretory phenotype production, contributes to atherosclerosis, postinfarction remodeling, diabetic complications, and the AKI-to-CKD transition.^140,141^ uPAR surface expression increases across multiple senescence triggers, and engineered CAR (chimeric antigen receptor) T cells directed against uPAR selectively ablate senescent cells in vitro and in vivo.^125^ Anti-uPAR CAR T cells reversed CCl_4_-induced and nonalcoholic steatohepatitis (NASH)–induced liver fibrosis,^125^ and in aged mice improved glucose tolerance and exercise capacity.^126^ These CAR T cells developed immunologic memory, persisting as long-lived cells that eliminate newly arising senescent cells over time,^126^ and most recently reversed aging-associated defects in intestinal stem cell function and barrier integrity.^142^

The cardiovascular relevance of senolytic immunotherapy extends beyond metabolic improvements. Senescent cells accumulate in atherosclerotic plaques and in the myocardium after ischemic injury, and genetic clearance of senescent cells attenuates atherosclerosis and improves cardiac function in preclinical models.^140,141^ Whether anti-uPAR CAR T cells can replicate these benefits by selectively targeting cardiovascular senescent cells has not been tested,^125,126,142^ and it is important to note that no cardiovascular or renal end points have been evaluated with this approach in any preclinical model to date.

The dominant risk is on-target, off-senescence toxicity: uPAR is expressed on activated monocytes, macrophages, and neutrophils (see section The Myeloid-Vascular Axis in Atherosclerosis), so anti-uPAR CAR T cells could deplete myeloid populations central to host defense, a concern not flagged in published preclinical studies^125,126,142^ but possibly understated in models that do not fully reflect the infectious and inflammatory challenges faced by CKM patients, many of whom are immunocompromised by diabetes, uremia, or immunosuppression. Mitigation strategies include expression-threshold gating (targeting the high uPAR characteristic of senescence and sparing moderate expressors), combinatorial antigen gating requiring a second senescence marker, and safety switches for CAR deactivation. All evidence remains preclinical, and Human translation faces manufacturing, cost, and regulatory barriers, although durable single-dose efficacy with immunologic memory substantially improves the risk-benefit profile over repeated-dose approaches.^126^

### Downstream Pathway Targeting

The intracellular cascades detailed in section Intracellular Signaling Modules of uPAR and suPAR identify pharmacologically tractable nodes organized from upstream (broadest effect) to terminal (most selective).

RAGE antagonists (azeliragon, FPS-ZM1) sit at the cascade apex across multiple cell types and have shown preclinical efficacy in diabetic complications, sepsis, and AKI.^143–145^ However, RAGE mediates physiological AGE clearance and pathogen recognition, raising concerns about chronic blockade. NOX2 occupies a similarly upstream position in podocytes,^72^ but its essential role in phagocytic host defense could limit the tolerability of systemic inhibition.^146,147^

Src transmits the ROS signal to TRPC6 mobilization in podocytes and mediates FAK-dependent dynamics in vascular cells, making it a convergence node. Dasatinib and selective FAK inhibitors are in clinical development for oncology,^148,149^ and Src inhibitors reduce proteinuria in suPAR-overexpressing mice (see section suPAR-Activated Signaling Cascades).^92,93^ However, repurposing for CKM syndrome requires achieving selectivity for pathological signaling while sparing physiological Src/FAK activity.

TRPC6 is the most downstream and podocyte-selective target: Gain-of-function *TRPC6* mutations cause familial FSGS,^91^ and inhibitors are in phase 2 testing for proteinuric CKD.^71,150^ As a terminal effector, TRPC6 inhibition offers the highest selectivity but the narrowest scope; it does not address myeloid priming, endothelial barrier disruption, or tubular ER stress. Conceptually distinct is the NF-κB/*PLAUR* loop (see section suPAR-Activated Signaling Cascades): targeting suPAR production at its source. Since rising suPAR reflects production rather than impaired clearance, NF-κB or JAK inhibitors could reduce systemic suPAR burden across tissues, with predictable immunosuppressive risk on chronic dosing.^91,151^

### Toward Integrated Therapeutic Strategies

The central argument of this review, that uPAR/suPAR signaling operates in distinct modes across disease contexts, has direct implications for therapeutic design: the optimal intervention depends on which signaling mode predominates (Table 2). In systemic -suPAR-dominant conditions (FSGS, sepsis-associated AKI, D2D3-mediated β cell injury), suPAR neutralization addresses the proximate cause and can be combined with TRPC6 or Src inhibition for synergistic podocyte protection. Atherosclerosis is more complex: suPAR primes circulating monocytes (see section The Myeloid-Vascular Axis in Atherosclerosis) while membrane uPAR on subendothelial macrophages drives plaque expansion; a systemic anti-uPAR antibody could intercept both arms, making atherosclerosis a compelling indication. In diabetic CKM, where both modes converge on RAGE and the D2D3-β cell axis creates a vicious cycle, multi-target combinations of suPAR neutralization with RAGE antagonism or *PLAUR* suppression may be necessary. Senolytic CAR T therapy complements all these strategies by eliminating the uPAR-positive senescent cells that feed the *PLAUR* amplification loop, addressing the cellular source of suPAR production.

Underpinning all these strategies is a critical translational prerequisite: patient stratification. The heterogeneous response to plasmapheresis in FSGS, the variable suPAR levels within CKM populations, and the fragment-specific pathobiology of D2D3 versus full-length suPAR all argue against a uniform approach. Companion diagnostics integrating suPAR fragment profiling, *PLAUR* genotype, APOL1 status, and disease phenotype will be essential to match patients to the therapeutic strategy most likely to benefit them.

## Outstanding Questions and Future Perspectives

### Molecular and Structural Priorities

The most consequential structural gap is the absence of high-resolution data for the suPAR–RAGE–αvβ3 ternary complex. Whether integrins serve as direct bridging adaptors or proximity facilitators whose clustering creates a permissive signaling platform remains unknown; cocrystal or cryo-EM structures would resolve this question and inform peptidomimetic inhibitor design.^14^ The biology of suPAR fragments also requires systematic investigation: the relative pathogenic potencies of full-length suPAR versus D2D3 across different target cells have not been compared head-to-head, and whether D1 removal exposes neoepitopes that alter receptor binding kinetics is unknown.^12^ If D2D3 is the dominant pathogenic species, fragment-specific antibodies and diagnostic assays become priority targets. Inflammatory glycosylation changes may generate suPAR glycoforms with altered receptor affinity or tissue selectivity; glycoproteomic comparison of healthy versus CKM suPAR could reveal targetable modifications. Dimeric suPAR species may alter receptor stoichiometry or signaling kinetics. More broadly, the CKM environment (hyperglycemia, dyslipidemia, uremia) could shift protease activity (full-length:D2D3 ratio), alter lipid raft dynamics,^148^ or glycate coreceptor interfaces; these represent a set of testable hypotheses connecting uPAR structural biology to its clinical epidemiology.

### Biological Priorities

The suPAR signaling cascade mapped in podocytes (see section suPAR-Activated Signaling Cascades) has not been systematically compared across other αvβ3-expressing cell types. Whether it is conserved (favoring upstream targets like NOX2) or diverges (as in tubular cells, where ER stress is the terminal effector) has direct therapeutic implications.^152^ The NF-κB/*PLAUR* feed-forward loop (see section Transcriptional Amplification of *PLAUR* Expression) has not been validated in CKM-relevant cell types; demonstrating its activity in myeloid cells or podocytes would establish it as a therapeutic target and reframe progressive suPAR elevation in CKD as actively driven.^90^ Whether suPAR and trained immunity are linked is unexplored but mechanistically plausible: suPAR could induce the epigenetic reprogramming (H3K4me3, H3K27ac at inflammatory loci) that defines trained immunity, but this has not been tested.^103^ The relative contributions of different tissue sources to suPAR (bone marrow myeloid cells versus adipose tissue macrophages versus Kupffer cells) remain poorly characterized; lineage-tracing and tissue-specific *PLAUR* knockout models would clarify targeting strategies. Finally, testing anti-uPAR CAR T cells in post-AKI models would simultaneously validate uPAR as a senescence marker in injured tubular epithelium and evaluate whether eliminating the cellular source of sustained inflammation can prevent the AKI-to-CKD transition.^125,126,142^

### Translational Priorities

Patient stratification is the most pressing translational need. An integrated diagnostic framework combining suPAR fragment profiling (full-length versus D2D3), *PLAUR* genotype, APOL1 risk status, and inflammatory biomarker panels could enable precision targeting of suPAR-directed therapies (Table 2). Development of fragment-specific immunoassays is a prerequisite. Clinical trial design faces specific challenges: end points must be disease-context-dependent (proteinuria for FSGS, major adverse cardiovascular event for atherosclerosis, glycemic control for D2D3-mediated β cell injury); target-engagement biomarkers demonstrating pathway inhibition in the relevant tissue compartment are needed; and the multi-hit pathogenesis model (see section Diabetes as a Modifier of suPAR Pathobiology) predicts that monotherapy may have modest effects, arguing for combination trials informed by the signaling hierarchy mapped in this review.

## Closing Perspective

This review has developed a framework in which a single receptor system, distinguished by its membrane-tethered versus soluble signaling modes, organizes local and systemic inflammatory programs across the organ systems that define CKM syndrome. The framework reframes suPAR not as a passive biomarker but as an active endocrine-like mediator extending uPAR’s signaling reach from the cell surface to distant organs. Filling the knowledge gaps outlined above, specifically the structural basis of the suPAR-RAGE–integrin complex, D2D3 fragment-specific biology, and patient stratification tools, would transform this framework from a conceptual model into a clinically actionable precision medicine platform. The tools to do so, including cryo-EM, single-cell spatial transcriptomics, glycoproteomics, and fragment-specific immunoassays, are now available; what is needed is their systematic application to CKM-relevant tissues and populations.

## ARTICLE INFORMATION

### Acknowledgments

The authors would like to acknowledge Dr Theresa Farhat for her feedback and editorial support.

### Disclosures

S.S. Hayek has patents filed for the use of suPAR (soluble urokinase plasminogen activator receptor) levels in the management of cardiovascular disease and the use of anti-suPAR therapies as a strategy for preventing and treating atherosclerosis (17/694,295 and 63/377,653). The other author reports no conflicts.

## References

[1] Hayek SS Sever S Ko YA Trachtman H Awad M Wadhwani S Altintas MM Wei C Hotton AL French AL. Soluble urokinase receptor and chronic kidney disease. N Engl J Med. 2015;373:1916–1925. doi: 10.1056/NEJMoa1506362 26539835 10.1056/NEJMoa1506362 PMC4701036

[2] Nikorowitsch J Borchardt T Appelbaum S Ojeda F Lackner KJ Schnabel RB Blankenberg S Zeller T Karakas M. Cardio-renal biomarker soluble urokinase-type plasminogen activator receptor is associated with cardiovascular death and myocardial infarction in patients with coronary artery disease independent of troponin, C-reactive protein, and renal function. J Am Heart Assoc. 2020;9:e015452. doi: 10.1161/JAHA.119.015452 32299288 10.1161/JAHA.119.015452 PMC7428542

[3] Stahl A Mueller BM. The urokinase-type plasminogen activator receptor, a GPI-linked protein, is localized in caveolae. J Cell Biol. 1995;129:335–344. doi: 10.1083/jcb.129.2.335 7721938 10.1083/jcb.129.2.335 PMC2199914

[4] Higazi AA Mazar A Wang J Quan N Griffin R Reilly R Henkin J Cines DB. Soluble human urokinase receptor is composed of two active units. J Biol Chem. 1997;272:5348–5353. doi: 10.1074/jbc.272.8.5348 9030610 10.1074/jbc.272.8.5348

[5] Tang H Kerins DM Hao Q Inagami T Vaughan DE. The urokinase-type plasminogen activator receptor mediates tyrosine phosphorylation of focal adhesion proteins and activation of mitogen-activated protein kinase in cultured endothelial cells. J Biol Chem. 1998;273:18268–18272. doi: 10.1074/jbc.273.29.18268 9660790 10.1074/jbc.273.29.18268

[6] Ferraris GM Sidenius N. Urokinase plasminogen activator receptor: a functional integrator of extracellular proteolysis, cell adhesion, and signal transduction. Semin Thromb Hemost. 2013;39:347–355. doi: 10.1055/s-0033-1334485 23532573 10.1055/s-0033-1334485

[7] Alfano D Franco P Stoppelli MP. Modulation of cellular function by the urokinase receptor signalling: a mechanistic view. Front Cell Dev Biol. 2022;10:818616. doi: 10.3389/fcell.2022.818616 35493073 10.3389/fcell.2022.818616 PMC9045800

[8] Resnati M Pallavicini I Wang JM Oppenheim J Serhan CN Romano M Blasi F. The fibrinolytic receptor for urokinase activates the G protein-coupled chemotactic receptor FPRL1/LXA4R. Proc Natl Acad Sci U S A. 2002;99:1359–1364. doi: 10.1073/pnas.022652999 11818541 10.1073/pnas.022652999 PMC122195

[9] Staubach S Hanisch FG. Lipid rafts: signaling and sorting platforms of cells and their roles in cancer. Expert Rev Proteomics. 2011;8:263–277. doi: 10.1586/epr.11.2 21501018 10.1586/epr.11.2

[10] Jhee JH Nam BY Lee CJ Park JT Han SH Kang SW Park S Yoo TH. Soluble urokinase-type plasminogen activator receptor, changes of 24-hour blood pressure, and progression of chronic kidney disease. J Am Heart Assoc. 2021;10:e017225. doi: 10.1161/JAHA.120.017225 33325248 10.1161/JAHA.120.017225 PMC7955457

[11] Hindy G Tyrrell DJ Vasbinder A Wei C Presswalla F Wang H Blakely P Ozel AB Graham S Holton GH; DBDS Consortium. Increased soluble urokinase plasminogen activator levels modulate monocyte function to promote atherosclerosis. J Clin Invest. 2022;132:e158788. doi: 10.1172/JCI158788 36194491 10.1172/JCI158788 PMC9754000

[12] Zhu K Mukherjee K Wei C Hayek SS Collins A Gu C Corapi K Altintas MM Wang Y Waikar SS. The D2D3 form of uPAR acts as an immunotoxin and may cause diabetes and kidney disease. Sci Transl Med. 2023;15:eabq6492. doi: 10.1126/scitranslmed.abq6492 37729431 10.1126/scitranslmed.abq6492

[13] Hayek SS Leaf DE Samman Tahhan A Raad M Sharma S Waikar SS Sever S Camacho A Wang X Dande RR. Soluble urokinase receptor and acute kidney injury. N Engl J Med. 2020;382:416–426. doi: 10.1056/NEJMoa1911481 31995687 10.1056/NEJMoa1911481 PMC7065830

[14] Kim EY Dryer SE. RAGE and αVβ3-integrin are essential for suPAR signaling in podocytes. Biochim Biophys Acta Mol Basis Dis. 2021;1867:166186. doi: 10.1016/j.bbadis.2021.166186 34166766 10.1016/j.bbadis.2021.166186 PMC8328937

[15] Selleri C Montuori N Ricci P Visconte V Baiano A Carriero MV Rotoli B Rossi G Ragno P. In vivo activity of the cleaved form of soluble urokinase receptor: a new hematopoietic stem/progenitor cell mobilizer. Cancer Res. 2006;66:10885–10890. doi: 10.1158/0008-5472.CAN-06-1311 17108125 10.1158/0008-5472.CAN-06-1311

[16] Montuori N Visconte V Rossi G Ragno P. Soluble and cleaved forms of the urokinase-receptor: degradation products or active molecules? Thromb Haemost. 2005;93:192–198. doi: 10.1160/TH04-09-0580 15711732 10.1160/TH04-09-0580

[17] Rasmussen LJH Petersen JEV Eugen-Olsen J. Soluble Urokinase Plasminogen Activator Receptor (suPAR) as a biomarker of systemic chronic inflammation. Front Immunol. 2021;12:780641. doi: 10.3389/fimmu.2021.780641 34925360 10.3389/fimmu.2021.780641 PMC8674945

[18] Alfano M Cinque P Giusti G Proietti S Nebuloni M Danese S D’Alessio S Genua M Portale F Lo Porto M. Full-length soluble urokinase plasminogen activator receptor down-modulates nephrin expression in podocytes. Sci Rep. 2015;5:13647. doi: 10.1038/srep13647 26380915 10.1038/srep13647 PMC4585377

[19] Nusshag C Wei C Hahm E Hayek SS Li J Samelko B Rupp C Szudarek R Speer C Kalble F. suPAR links a dysregulated immune response to tissue inflammation and sepsis-induced acute kidney injury. JCI Insight. 2023;8:e165740. doi: 10.1172/jci.insight.165740 37036003 10.1172/jci.insight.165740 PMC10132159

[20] Wei Y Czekay RP Robillard L Kugler MC Zhang F Kim KK Xiong JP Humphries MJ Chapman HA. Regulation of alpha5beta1 integrin conformation and function by urokinase receptor binding. J Cell Biol. 2005;168:501–511. doi: 10.1083/jcb.200404112 15684035 10.1083/jcb.200404112 PMC2171741

[21] Blasi F Sidenius N. The urokinase receptor: focused cell surface proteolysis, cell adhesion and signaling. FEBS Lett. 2010;584:1923–1930. doi: 10.1016/j.febslet.2009.12.039 20036661 10.1016/j.febslet.2009.12.039

[22] Wang B Wang J Qi C Gao C Wang Y Zan Y Tan Y Wu Z Jiang J Suo J. Soluble urokinase plasminogen activator receptor promotes endoplasmic reticulum stress and apoptosis susceptibility through RAGE in sepsis acute kidney injury. Mol Med. 2025;31:296. doi: 10.1186/s10020-025-01352-w 41013175 10.1186/s10020-025-01352-w PMC12465643

[23] Kim EY Dryer SE. Role of formyl peptide receptors and β-arrestin-1 in suPAR Signal transduction in mouse podocytes: interactions with αVβ3-integrin. Cells. 2024;13:172. doi: 10.3390/cells13020172 38247863 10.3390/cells13020172 PMC10814688

[24] Elwakiel A Gupta D Rana R Manoharan J Al-Dabet MM Ambreen S Fatima S Zimmermann S Mathew A Li Z. Factor XII signaling via uPAR-integrin β1 axis promotes tubular senescence in diabetic kidney disease. Nat Commun. 2024;15:7963. doi: 10.1038/s41467-024-52214-8 39261453 10.1038/s41467-024-52214-8 PMC11390906

[25] Llinas P Du Le MH Gardsvoll H Dano K Ploug M Gilquin B Stura EA Menez A. Crystal structure of the human urokinase plasminogen activator receptor bound to an antagonist peptide. EMBO J. 2005;24:1655–1663. doi: 10.1038/sj.emboj.7600635 15861141 10.1038/sj.emboj.7600635 PMC1142576

[26] Mertens HD Kjaergaard M Mysling S Gardsvoll H Jorgensen TJ Svergun DI Ploug M. A flexible multidomain structure drives the function of the Urokinase-Type Plasminogen Activator Receptor (uPAR). J Biol Chem. 2012;287:34304–34315. doi: 10.1074/jbc.M112.398404 22896701 10.1074/jbc.M112.398404 PMC3464537

[27] Liu M Lin L Hoyer-Hansen G Ploug M Li H Jiang L Yuan C Li J Huang M. Crystal structure of the unoccupied murine Urokinase-Type Plasminogen Activator Receptor (uPAR) reveals a tightly packed DII-DIII unit. FEBS Lett. 2019;593:1236–1247. doi: 10.1002/1873-3468.13397 31044429 10.1002/1873-3468.13397

[28] Yu S Sui Y Wang J Li Y Li H Cao Y Chen L Jiang L Yuan C Huang M. Crystal structure and cellular functions of uPAR dimer. Nat Commun. 2022;13:1665. doi: 10.1038/s41467-022-29344-y 35351875 10.1038/s41467-022-29344-y PMC8964761

[29] Leth JM Mertens HDT Leth-Espensen KZ Jorgensen TJD Ploug M. Did evolution create a flexible ligand-binding cavity in the urokinase receptor through deletion of a plesiotypic disulfide bond? J Biol Chem. 2019;294:7403–7418. doi: 10.1074/jbc.RA119.007847 30894413 10.1074/jbc.RA119.007847 PMC6509485

[30] Kjoller L Hall A. Rac mediates cytoskeletal rearrangements and increased cell motility induced by urokinase-type plasminogen activator receptor binding to vitronectin. J Cell Biol. 2001;152:1145–1157. doi: 10.1083/jcb.152.6.1145 11257116 10.1083/jcb.152.6.1145 PMC2199201

[31] Madsen CD Ferraris GM Andolfo A Cunningham O Sidenius N. uPAR-induced cell adhesion and migration: vitronectin provides the key. J Cell Biol. 2007;177:927–939. doi: 10.1083/jcb.200612058 17548516 10.1083/jcb.200612058 PMC2064291

[32] Gardsvoll H Kjaergaard M Jacobsen B Kriegbaum MC Huang M Ploug M. Mimicry of the regulatory role of urokinase in lamellipodia formation by introduction of a non-native interdomain disulfide bond in its receptor. J Biol Chem. 2011;286:43515–43526. doi: 10.1074/jbc.M111.300020 22025616 10.1074/jbc.M111.300020 PMC3234840

[33] Bottcher RT Fassler R. Membrane tension drives ligand-independent integrin signaling. EMBO J. 2014;33:2439–2441. doi: 10.15252/embj.201489886 25230934 10.15252/embj.201489886 PMC4283401

[34] Degryse B Resnati M Czekay RP Loskutoff DJ Blasi F. Domain 2 of the urokinase receptor contains an integrin-interacting epitope with intrinsic signaling activity: generation of a new integrin inhibitor. J Biol Chem. 2005;280:24792–24803. doi: 10.1074/jbc.M413954200 15863511 10.1074/jbc.M413954200

[35] Wilhelm OG Wilhelm S Escott GM Lutz V Magdolen V Schmitt M Rifkin DB Wilson EL Graeff H Brunner G. Cellular glycosylphosphatidylinositol-specific phospholipase D regulates urokinase receptor shedding and cell surface expression. J Cell Physiol. 1999;180:225–235. doi: 10.1002/(SICI)1097-4652(199908)180:2<225::AID-JCP10>3.0.CO;2-2 10395292 10.1002/(SICI)1097-4652(199908)180:2<225::AID-JCP10>3.0.CO;2-2

[36] O’Brien KD Pineda C Chiu WS Bowen R Deeg MA. Glycosylphosphatidylinositol-specific phospholipase D is expressed by macrophages in human atherosclerosis and colocalizes with oxidation epitopes. Circulation. 1999;99:2876–2882. doi: 10.1161/01.cir.99.22.2876 10359731 10.1161/01.cir.99.22.2876

[37] Kurtz TA Fineberg NS Considine RV Deeg MA. Insulin resistance is associated with increased serum levels of glycosylphosphatidylinositol-specific phospholipase D. Metabolism. 2004;53:138–139. doi: 10.1016/j.metabol.2003.09.004 14767861 10.1016/j.metabol.2003.09.004

[38] van Veen M Matas-Rico E van de Wetering K Leyton-Puig D Kedziora KM De Lorenzi V Stijf-Bultsma Y van den Broek B Jalink K Sidenius N. Negative regulation of urokinase receptor activity by a GPI-specific phospholipase C in breast cancer cells. Elife. 2017;6:e23649. doi: 10.7554/eLife.23649 28849762 10.7554/eLife.23649 PMC5576486

[39] Ploug M Eriksen J Plesner T Hansen NE Dano K. A soluble form of the glycolipid-anchored receptor for urokinase-type plasminogen activator is secreted from peripheral blood leukocytes from patients with paroxysmal nocturnal hemoglobinuria. Eur J Biochem. 1992;208:397–404. doi: 10.1111/j.1432-1033.1992.tb17200.x 1325906 10.1111/j.1432-1033.1992.tb17200.x

[40] Sidenius N Sier CF Blasi F. Shedding and cleavage of the urokinase receptor (uPAR): identification and characterisation of uPAR fragments in vitro and in vivo. FEBS Lett. 2000;475:52–56. doi: 10.1016/s0014-5793(00)01624-0 10854857 10.1016/s0014-5793(00)01624-0

[41] Montuori N Carriero MV Salzano S Rossi G Ragno P. The cleavage of the urokinase receptor regulates its multiple functions. J Biol Chem. 2002;277:46932–46939. doi: 10.1074/jbc.M207494200 12297505 10.1074/jbc.M207494200

[42] Pliyev BK. Activated human neutrophils rapidly release the chemotactically active D2D3 form of the Urokinase-Type Plasminogen Activator Receptor (uPAR/CD87). Mol Cell Biochem. 2009;321:111–122. doi: 10.1007/s11010-008-9925-z 18830568 10.1007/s11010-008-9925-z

[43] Thuno M Macho B Eugen-Olsen J. suPAR. The molecular crystal ball. Dis Markers. 2009;27:157–172. doi: 10.3233/DMA-2009-0657 19893210 10.3233/DMA-2009-0657 PMC3835059

[44] Montuori N Bifulco K Carriero MV La Penna C Visconte V Alfano D Pesapane A Rossi FW Salzano S Rossi G. The cross-talk between the urokinase receptor and fMLP receptors regulates the activity of the CXCR4 chemokine receptor. Cell Mol Life Sci. 2011;68:2453–2467. doi: 10.1007/s00018-010-0564-7 20972812 10.1007/s00018-010-0564-7 PMC11114667

[45] Simons K Ikonen E. Functional rafts in cell membranes. Nature. 1997;387:569–572. doi: 10.1038/42408 9177342 10.1038/42408

[46] Pike LJ. Lipid rafts: bringing order to chaos. J Lipid Res. 2003;44:655–667. doi: 10.1194/jlr.R200021-JLR200 12562849 10.1194/jlr.R200021-JLR200

[47] Cunningham O Andolfo A Santovito ML Iuzzolino L Blasi F Sidenius N. Dimerization controls the lipid raft partitioning of uPAR/CD87 and regulates its biological functions. EMBO J. 2003;22:5994–6003. doi: 10.1093/emboj/cdg588 14609946 10.1093/emboj/cdg588 PMC275445

[48] Ferraris GM Schulte C Buttiglione V De Lorenzi V Piontini A Galluzzi M Podesta A Madsen CD Sidenius N. The interaction between uPAR and vitronectin triggers ligand-independent adhesion signalling by integrins. EMBO J. 2014;33:2458–2472. doi: 10.15252/embj.201387611 25168639 10.15252/embj.201387611 PMC4283405

[49] Hayek SS Koh KH Grams ME Wei C Ko YA Li J Samelko B Lee H Dande RR Lee HW. A tripartite complex of suPAR, APOL1 risk variants and αvβ3 integrin on podocytes mediates chronic kidney disease. Nat Med. 2017;23:945–953. doi: 10.1038/nm.4362 28650456 10.1038/nm.4362 PMC6019326

[50] Jain M Chauhan AK. Role of integrins in modulating smooth muscle cell plasticity and vascular remodeling: from expression to therapeutic implications. Cells. 2022;11:646. doi: 10.3390/cells11040646 35203297 10.3390/cells11040646 PMC8870356

[51] Moraes JA Frony AC Dias AM Renovato-Martins M Rodrigues G Marcinkiewicz C Assreuy J Barja-Fidalgo C. Alpha1beta1 and integrin-linked kinase interact and modulate angiotensin II effects in vascular smooth muscle cells. Atherosclerosis. 2015;243:477–485. doi: 10.1016/j.atherosclerosis.2015.09.026 26520903 10.1016/j.atherosclerosis.2015.09.026

[52] Kiyan J Kiyan R Haller H Dumler I. Urokinase-induced signaling in human vascular smooth muscle cells is mediated by PDGFR-beta. EMBO J. 2005;24:1787–1797. doi: 10.1038/sj.emboj.7600669 15889147 10.1038/sj.emboj.7600669 PMC1142599

[53] May AE Kanse SM Lund LR Gisler RH Imhof BA Preissner KT. Urokinase receptor (CD87) regulates leukocyte recruitment via beta 2 integrins in vivo. J Exp Med. 1998;188:1029–1037. doi: 10.1084/jem.188.6.1029 9743521 10.1084/jem.188.6.1029 PMC2212528

[54] May AE Neumann FJ Schomig A Preissner KT. VLA-4 (alpha(4)beta(1)) engagement defines a novel activation pathway for beta(2) integrin-dependent leukocyte adhesion involving the urokinase receptor. Blood. 2000;96:506–513. doi: 10.1182/blood.V96.2.506 10887112

[55] Zhang H Colman RW Sheng N. Regulation of CD11b/CD18 (Mac-1) adhesion to fibrinogen by urokinase receptor (uPAR). Inflamm Res. 2003;52:86–93. doi: 10.1007/s000110300006 12665127 10.1007/s000110300006

[56] Wei Y Waltz DA Rao N Drummond RJ Rosenberg S Chapman HA. Identification of the urokinase receptor as an adhesion receptor for vitronectin. J Biol Chem. 1994;269:32380–32388. doi: 10.1016/S0021-9258(18)31646-6 7528215

[57] Wei Y Eble JA Wang Z Kreidberg JA Chapman HA. Urokinase receptors promote beta1 integrin function through interactions with integrin alpha3beta1. Mol Biol Cell. 2001;12:2975–2986. doi: 10.1091/mbc.12.10.2975 11598185 10.1091/mbc.12.10.2975 PMC60149

[58] Gardsvoll H Ploug M. Mapping of the vitronectin-binding site on the urokinase receptor: involvement of a coherent receptor interface consisting of residues from both domain I and the flanking interdomain linker region. J Biol Chem. 2007;282:13561–13572. doi: 10.1074/jbc.M610184200 17355965 10.1074/jbc.M610184200

[59] Lopez-Alemany R Redondo JM Nagamine Y Munoz-Canoves P. Plasminogen activator inhibitor type-1 inhibits insulin signaling by competing with alphavbeta3 integrin for vitronectin binding. Eur J Biochem. 2003;270:814–821. doi: 10.1046/j.1432-1033.2003.03453.x 12603314 10.1046/j.1432-1033.2003.03453.x

[60] Czekay RP Aertgeerts K Curriden SA Loskutoff DJ. Plasminogen activator inhibitor-1 detaches cells from extracellular matrices by inactivating integrins. J Cell Biol. 2003;160:781–791. doi: 10.1083/jcb.200208117 12615913 10.1083/jcb.200208117 PMC2173358

[61] Jo M Thomas KS O’Donnell DM Gonias SL. Epidermal growth factor receptor-dependent and -independent cell-signaling pathways originating from the urokinase receptor. J Biol Chem. 2003;278:1642–1646. doi: 10.1074/jbc.M210877200 12426305 10.1074/jbc.M210877200

[62] Liu D Aguirre Ghiso J Estrada Y Ossowski L. EGFR is a transducer of the urokinase receptor initiated signal that is required for in vivo growth of a human carcinoma. Cancer Cell. 2002;1:445–457. doi: 10.1016/s1535-6108(02)00072-7 12124174 10.1016/s1535-6108(02)00072-7

[63] Jo M Thomas KS Marozkina N Amin TJ Silva CM Parsons SJ Gonias SL. Dynamic assembly of the urokinase-type plasminogen activator signaling receptor complex determines the mitogenic activity of urokinase-type plasminogen activator. J Biol Chem. 2005;280:17449–17457. doi: 10.1074/jbc.M413141200 15728176 10.1074/jbc.M413141200

[64] Abu-Ali S Fotovati A Shirasuna K. Tyrosine-kinase inhibition results in EGFR clustering at focal adhesions and consequent exocytosis in uPAR down-regulated cells of head and neck cancers. Mol Cancer. 2008;7:47. doi: 10.1186/1476-4598-7-47 18519000 10.1186/1476-4598-7-47 PMC2464604

[65] Jo M Thomas KS Takimoto S Gaultier A Hsieh EH Lester RD Gonias SL. Urokinase receptor primes cells to proliferate in response to epidermal growth factor. Oncogene. 2007;26:2585–2594. doi: 10.1038/sj.onc.1210066 17043637 10.1038/sj.onc.1210066

[66] Wei Y Lukashev M Simon DI Bodary SC Rosenberg S Doyle MV Chapman HA. Regulation of integrin function by the urokinase receptor. Science. 1996;273:1551–1555. doi: 10.1126/science.273.5281.1551 8703217 10.1126/science.273.5281.1551

[67] Chuah YK Basir R Talib H Tie TH Nordin N. Receptor for advanced glycation end products and its involvement in inflammatory diseases. Int J Inflam. 2013;2013:403460. doi: 10.1155/2013/403460 24102034 10.1155/2013/403460 PMC3786507

[68] Kierdorf K Fritz G. RAGE regulation and signaling in inflammation and beyond. J Leukoc Biol. 2013;94:55–68. doi: 10.1189/jlb.1012519 23543766 10.1189/jlb.1012519

[69] Wei C Moller CC Altintas MM Li J Schwarz K Zacchigna S Xie L Henger A Schmid H Rastaldi MP. Modification of kidney barrier function by the urokinase receptor. Nat Med. 2008;14:55–63. doi: 10.1038/nm1696 18084301 10.1038/nm1696

[70] Wei C El Hindi S Li J Fornoni A Goes N Sageshima J Maiguel D Karumanchi SA Yap HK Saleem M. Circulating urokinase receptor as a cause of focal segmental glomerulosclerosis. Nat Med. 2011;17:952–960. doi: 10.1038/nm.2411 21804539 10.1038/nm.2411 PMC4089394

[71] Kim EY Roshanravan H Dryer SE. Changes in podocyte TRPC channels evoked by plasma and sera from patients with recurrent FSGS and by putative glomerular permeability factors. Biochim Biophys Acta Mol Basis Dis. 2017;1863:2342–2354. doi: 10.1016/j.bbadis.2017.06.010 28629718 10.1016/j.bbadis.2017.06.010 PMC5557291

[72] Kim EY Hassanzadeh Khayyat N Dryer SE. Mechanisms underlying modulation of podocyte TRPC6 channels by suPAR: role of NADPH oxidases and Src family tyrosine kinases. Biochim Biophys Acta Mol Basis Dis. 2018;1864:3527–3536. doi: 10.1016/j.bbadis.2018.08.007 30293571 10.1016/j.bbadis.2018.08.007 PMC6219472

[73] Koh KH Cao Y Mangos S Tardi NJ Dande RR Lee HW Samelko B Altintas MM Schmitz VP Lee H. Nonimmune cell-derived ICOS ligand functions as a renoprotective αvβ3 integrin-selective antagonist. J Clin Invest. 2019;129:1713–1726. doi: 10.1172/JCI123386 30747722 10.1172/JCI123386 PMC6436851

[74] Staeck O Slowinski T Lieker I Wu K Rudolph B Schmidt D Brakemeier S Neumayer HH Wei C Reiser J. Recurrent primary focal segmental glomerulosclerosis managed with intensified plasma exchange and concomitant monitoring of soluble urokinase-type plasminogen activator receptor-mediated podocyte β3-integrin activation. Transplantation. 2015;99:2593–2597. doi: 10.1097/TP.0000000000000914 26371597 10.1097/TP.0000000000000914 PMC4900174

[75] Li L Chen K Xiang Y Yoshimura T Su S Zhu J Bian XW Wang JM. New development in studies of formyl-peptide receptors: critical roles in host defense. J Leukoc Biol. 2016;99:425–435. doi: 10.1189/jlb.2RI0815-354RR 26701131 10.1189/jlb.2RI0815-354RR PMC4750370

[76] Siegel ER Croze RH Fang X Matthay MA Gotts JE. Inhibition of the lipoxin A4 and resolvin D1 receptor impairs host response to acute lung injury caused by pneumococcal pneumonia in mice. Am J Physiol Lung Cell Mol Physiol. 2021;320:L1085–L1092. doi: 10.1152/ajplung.00046.2021 33822656 10.1152/ajplung.00046.2021 PMC8285627

[77] Asahina Y Wurtz NR Arakawa K Carson N Fujii K Fukuchi K Garcia R Hsu MY Ishiyama J Ito B. Discovery of BMS-986235/LAR-1219: a potent Formyl Peptide Receptor 2 (FPR2) selective agonist for the prevention of heart failure. J Med Chem. 2020;63:9003–9019. doi: 10.1021/acs.jmedchem.9b02101 32407089 10.1021/acs.jmedchem.9b02101

[78] Lind S Dahlgren C Holmdahl R Olofsson P Forsman H. Functional selective FPR1 signaling in favor of an activation of the neutrophil superoxide generating NOX2 complex. J Leukoc Biol. 2021;109:1105–1120. doi: 10.1002/JLB.2HI0520-317R 33040403 10.1002/JLB.2HI0520-317R PMC8246850

[79] Gao JL Chen H Filie JD Kozak CA Murphy PM. Differential expansion of the N-formylpeptide receptor gene cluster in human and mouse. Genomics. 1998;51:270–276. doi: 10.1006/geno.1998.5376 9722950 10.1006/geno.1998.5376

[80] Nguyen DH Hussaini IM Gonias SL. Binding of urokinase-type plasminogen activator to its receptor in MCF-7 cells activates extracellular signal-regulated kinase 1 and 2 which is required for increased cellular motility. J Biol Chem. 1998;273:8502–8507. doi: 10.1074/jbc.273.14.8502 9525964 10.1074/jbc.273.14.8502

[81] Larusch GA Merkulova A Mahdi F Shariat-Madar Z Sitrin RG Cines DB Schmaier AH. Domain 2 of uPAR regulates single-chain urokinase-mediated angiogenesis through β1-integrin and VEGFR2. Am J Physiol Heart Circ Physiol. 2013;305:H305–H320. doi: 10.1152/ajpheart.00110.2013 23709605 10.1152/ajpheart.00110.2013 PMC3742872

[82] Schreiber TD Steinl C Essl M Abele H Geiger K Muller CA Aicher WK Klein G. The integrin alpha9beta1 on hematopoietic stem and progenitor cells: involvement in cell adhesion, proliferation and differentiation. Haematologica. 2009;94:1493–1501. doi: 10.3324/haematol.2009.006072 19608669 10.3324/haematol.2009.006072 PMC2770959

[83] del Pozo MA Alderson NB Kiosses WB Chiang HH Anderson RG Schwartz MA. Integrins regulate Rac targeting by internalization of membrane domains. Science. 2004;303:839–842. doi: 10.1126/science.1092571 14764880 10.1126/science.1092571

[84] Smith HW Marra P Marshall CJ. uPAR promotes formation of the p130Cas-Crk complex to activate Rac through DOCK180. J Cell Biol. 2008;182:777–790. doi: 10.1083/jcb.200712050 18725541 10.1083/jcb.200712050 PMC2518715

[85] Kjoller L. The urokinase plasminogen activator receptor in the regulation of the actin cytoskeleton and cell motility. Biol Chem. 2002;383:5–19. doi: 10.1515/BC.2002.002 11928822 10.1515/BC.2002.002

[86] Margheri F Luciani C Taddei ML Giannoni E Laurenzana A Biagioni A Chilla A Chiarugi P Fibbi G Del Rosso M. The receptor for Urokinase-Plasminogen Activator (uPAR) controls plasticity of cancer cell movement in mesenchymal and amoeboid migration style. Oncotarget. 2014;5:1538–1553. doi: 10.18632/oncotarget.1754 24681666 10.18632/oncotarget.1754 PMC4039230

[87] Dumler I Weis A Mayboroda OA Maasch C Jerke U Haller H Gulba DC. The Jak/Stat pathway and urokinase receptor signaling in human aortic vascular smooth muscle cells. J Biol Chem. 1998;273:315–321. doi: 10.1074/jbc.273.1.315 9417082 10.1074/jbc.273.1.315

[88] Gonias SL. Plasminogen activator receptor assemblies in cell signaling, innate immunity, and inflammation. Am J Physiol Cell Physiol. 2021;321:C721–C734. doi: 10.1152/ajpcell.00269.2021 34406905 10.1152/ajpcell.00269.2021 PMC8560384

[89] Wang Y Dang J Wang H Allgayer H Murrell GA Boyd D. Identification of a novel nuclear factor-kappaB sequence involved in expression of urokinase-type plasminogen activator receptor. Eur J Biochem. 2000;267:3248–3254. doi: 10.1046/j.1432-1327.2000.01350.x 10824110 10.1046/j.1432-1327.2000.01350.x

[90] Chew-Harris J Appleby S Richards AM Troughton RW Pemberton CJ. Analytical, biochemical and clearance considerations of Soluble Urokinase Plasminogen Activator Receptor (suPAR) in healthy individuals. Clin Biochem. 2019;69:36–44. doi: 10.1016/j.clinbiochem.2019.05.010 31129182 10.1016/j.clinbiochem.2019.05.010

[91] Staruschenko A Ma R Palygin O Dryer SE. Ion channels and channelopathies in glomeruli. Physiol Rev. 2023;103:787–854. doi: 10.1152/physrev.00013.2022 36007181 10.1152/physrev.00013.2022 PMC9662803

[92] Wei C Li J Adair BD Zhu K Cai J Merchant M Samelko B Liao Z Koh KH Tardi NJ. uPAR isoform 2 forms a dimer and induces severe kidney disease in mice. J Clin Invest. 2019;129:1946–1959. doi: 10.1172/JCI124793 30730305 10.1172/JCI124793 PMC6486353

[93] AboEl-Magd GH Mabrouk MM. Soluble urokinase-type plasminogen activator receptor as a measure of treatment response in acute exacerbation of COPD. J Bras Pneumol. 2018;44:36–41. doi: 10.1590/S1806-37562017000000151 29538541 10.1590/S1806-37562017000000151 PMC6104538

[94] Kim EY Dryer SE. TRPC6 effects on albumin permeation, nephrin shedding, and apoptosis in podocytes: role of calcineurin and metalloproteases. Physiol Rep. 2025;13:e70614. doi: 10.14814/phy2.70614 41165237 10.14814/phy2.70614 PMC12573276

[95] Rajagopalan S Meng XP Ramasamy S Harrison DG Galis ZS. Reactive oxygen species produced by macrophage-derived foam cells regulate the activity of vascular matrix metalloproteinases in vitro. Implications for atherosclerotic plaque stability. J Clin Invest. 1996;98:2572–2579. doi: 10.1172/JCI119076 8958220 10.1172/JCI119076 PMC507715

[96] Galis ZS Sukhova GK Lark MW Libby P. Increased expression of matrix metalloproteinases and matrix degrading activity in vulnerable regions of human atherosclerotic plaques. J Clin Invest. 1994;94:2493–2503. doi: 10.1172/JCI117619 7989608 10.1172/JCI117619 PMC330083

[97] Reiser J Altintas MM. Podocytes. F1000Res. 2016;5:F1000 Faculty Rev–F1000 Faculty 114. doi: 10.12688/f1000research.7255.1

[98] Veron D Villegas G Aggarwal PK Bertuccio C Jimenez J Velazquez H Reidy K Abrahamson DR Moeckel G Kashgarian M. Acute podocyte Vascular Endothelial Growth Factor (VEGF-A) knockdown disrupts alphaVbeta3 integrin signaling in the glomerulus. PLoS One. 2012;7:e40589. doi: 10.1371/journal.pone.0040589 22808199 10.1371/journal.pone.0040589 PMC3396653

[99] Kim EY Anderson M Wilson C Hagmann H Benzing T Dryer SE. NOX2 interacts with podocyte TRPC6 channels and contributes to their activation by diacylglycerol: essential role of podocin in formation of this complex. Am J Physiol Cell Physiol. 2013;305:C960–C971. doi: 10.1152/ajpcell.00191.2013 23948707 10.1152/ajpcell.00191.2013

[100] Feng D DuMontier C Pollak MR. Mechanical challenges and cytoskeletal impairments in focal segmental glomerulosclerosis. Am J Physiol Renal Physiol. 2018;314:F921–F925. doi: 10.1152/ajprenal.00641.2017 29363327 10.1152/ajprenal.00641.2017 PMC6031906

[101] Sitrin RG Todd RF III Albrecht E Gyetko MR. The urokinase receptor (CD87) facilitates CD11b/CD18-mediated adhesion of human monocytes. J Clin Invest. 1996;97:1942–1951. doi: 10.1172/JCI118626 8621779 10.1172/JCI118626 PMC507264

[102] Orlova VV Choi EY Xie C Chavakis E Bierhaus A Ihanus E Ballantyne CM Gahmberg CG Bianchi ME Nawroth PP. A novel pathway of HMGB1-mediated inflammatory cell recruitment that requires Mac-1-integrin. EMBO J. 2007;26:1129–1139. doi: 10.1038/sj.emboj.7601552 17268551 10.1038/sj.emboj.7601552 PMC1852832

[103] Netea MG Joosten LA Latz E Mills KH Natoli G Stunnenberg HG O’Neill LA Xavier RJ. Trained immunity: a program of innate immune memory in health and disease. Science. 2016;352:aaf1098. doi: 10.1126/science.aaf1098 27102489 10.1126/science.aaf1098 PMC5087274

[104] Saeed S Quintin J Kerstens HH Rao NA Aghajanirefah A Matarese F Cheng SC Ratter J Berentsen K van der Ent MA. Epigenetic programming of monocyte-to-macrophage differentiation and trained innate immunity. Science. 2014;345:1251086. doi: 10.1126/science.1251086 25258085 10.1126/science.1251086 PMC4242194

[105] Makarova AM Lebedeva TV Nassar T Higazi AA Xue J Carinato ME Bdeir K Cines DB Stepanova V. Urokinase-type Plasminogen Activator (uPA) induces pulmonary microvascular endothelial permeability through low density Lipoprotein Receptor-related Protein (LRP)-dependent activation of endothelial nitric-oxide synthase. J Biol Chem. 2011;286:23044–23053. doi: 10.1074/jbc.M110.210195 21540184 10.1074/jbc.M110.210195 PMC3123072

[106] Penkov D Beloglazova I Parfyonova Y. Endothelial-specific enhancer as a Cis Element OF PLAUR regulation by TNF-alpha, IL-1beta, and VEGF. Curr Pharm Des. 2024;30:1630–1640. doi: 10.2174/0113816128296376240424072322 38715331 10.2174/0113816128296376240424072322

[107] Angelini DJ Hyun SW Grigoryev DN Garg P Gong P Singh IS Passaniti A Hasday JD Goldblum SE. TNF-alpha increases tyrosine phosphorylation of vascular endothelial cadherin and opens the paracellular pathway through fyn activation in human lung endothelia. Am J Physiol Lung Cell Mol Physiol. 2006;291:L1232–L1245. doi: 10.1152/ajplung.00109.2006 16891393 10.1152/ajplung.00109.2006

[108] Broermann A Winderlich M Block H Frye M Rossaint J Zarbock A Cagna G Linnepe R Schulte D Nottebaum AF. Dissociation of VE-PTP from VE-cadherin is required for leukocyte extravasation and for VEGF-induced vascular permeability in vivo. J Exp Med. 2011;208:2393–2401. doi: 10.1084/jem.20110525 22025303 10.1084/jem.20110525 PMC3256962

[109] Larmann J Jurk K Janssen H Muller M Herzog C Lorenz A Schmitz M Nofer JR Theilmeier G. Hepatic Overexpression of Soluble Urokinase Receptor (uPAR) suppresses diet-induced atherosclerosis in Low-Density Lipoprotein Receptor-Deficient (LDLR-/-) Mice. PLoS One. 2015;10:e0131854. doi: 10.1371/journal.pone.0131854 26313756 10.1371/journal.pone.0131854 PMC4551736

[110] Ramirez GA Coletto LA Sciorati C Bozzolo EP Manunta P Rovere-Querini P Manfredi AA. Ion channels and transporters in inflammation: special focus on TRP channels and TRPC6. Cells. 2018;7:70. doi: 10.3390/cells7070070 29973568 10.3390/cells7070070 PMC6070975

[111] Damann N Owsianik G Li S Poll C Nilius B. The calcium-conducting ion channel transient receptor potential canonical 6 is involved in macrophage inflammatory protein-2-induced migration of mouse neutrophils. Acta Physiol (Oxf). 2009;195:3–11. doi: 10.1111/j.1748-1716.2008.01918.x 18983454 10.1111/j.1748-1716.2008.01918.x

[112] Lenin R Maria MS Agrawal M Balasubramanyam J Mohan V Balasubramanyam M. Amelioration of glucolipotoxicity-induced endoplasmic reticulum stress by a “chemical chaperone” in human THP-1 monocytes. Exp Diabetes Res. 2012;2012:356487. doi: 10.1155/2012/356487 22550476 10.1155/2012/356487 PMC3328920

[113] Haraldsson B Nystrom J Deen WM. Properties of the glomerular barrier and mechanisms of proteinuria. Physiol Rev. 2008;88:451–487. doi: 10.1152/physrev.00055.2006 18391170 10.1152/physrev.00055.2006

[114] Haraldsson B Jeansson M. Glomerular filtration barrier. Curr Opin Nephrol Hypertens. 2009;18:331–335. doi: 10.1097/MNH.0b013e32832c9dba 19458528 10.1097/MNH.0b013e32832c9dba

[115] Chang JW Pardo V Sageshima J Chen L Tsai HL Reiser J Wei C Ciancio G Burke GW III Fornoni A. Podocyte foot process effacement in postreperfusion allograft biopsies correlates with early recurrence of proteinuria in focal segmental glomerulosclerosis. Transplantation. 2012;93:1238–1244. doi: 10.1097/TP.0b013e318250234a 22499148 10.1097/TP.0b013e318250234a PMC3432300

[116] Campbell KN Tumlin JA. Protecting podocytes: a key target for therapy of focal segmental glomerulosclerosis. Am J Nephrol. 2018;47(Suppl 1):14–29. doi: 10.1159/000481634 29852493 10.1159/000481634 PMC6589822

[117] Alachkar N Wei C Arend LJ Jackson AM Racusen LC Fornoni A Burke G Rabb H Kakkad K Reiser J. Podocyte effacement closely links to suPAR levels at time of posttransplantation focal segmental glomerulosclerosis occurrence and improves with therapy. Transplantation. 2013;96:649–656. doi: 10.1097/TP.0b013e31829eda4f 23842190 10.1097/TP.0b013e31829eda4f PMC4026282

[118] Gomez H Ince C De Backer D Pickkers P Payen D Hotchkiss J Kellum JA. A unified theory of sepsis-induced acute kidney injury: inflammation, microcirculatory dysfunction, bioenergetics, and the tubular cell adaptation to injury. Shock. 2014;41:3–11. doi: 10.1097/SHK.0000000000000052 10.1097/SHK.0000000000000052 PMC3918942 24346647

[119] Liu YT Zhang H Duan SB Wang JW Chen H Zhan M Zhang W Li AM Liu Y Yang Y. Mitofusin2 ameliorated endoplasmic reticulum stress and mitochondrial reactive oxygen species through maintaining mitochondria-associated endoplasmic reticulum membrane integrity in cisplatin-induced acute kidney injury. Antioxid Redox Signal. 2024;40:16–39. doi: 10.1089/ars.2022.0178 37053105 10.1089/ars.2022.0178

[120] Jin H Yang Y Zhu X Zhou Y Xu Y Li J Qi C Shao X Wu J Wu S. DDRGK1-mediated ER-phagy attenuates acute kidney injury through ER-stress and apoptosis. Cell Death Dis. 2024;15:63. doi: 10.1038/s41419-024-06449-4 38233375 10.1038/s41419-024-06449-4 PMC10794694

[121] Cao Y Chen X Zhu Z Luo Z Hao Y Yang X Feng J Zhang Z Hu J Jian Y. STING contributes to lipopolysaccharide-induced tubular cell inflammation and pyroptosis by activating endoplasmic reticulum stress in acute kidney injury. Cell Death Dis. 2024;15:217. doi: 10.1038/s41419-024-06600-1 38485717 10.1038/s41419-024-06600-1 PMC10940292

[122] Gao P Yang W Sun L. Mitochondria-Associated Endoplasmic Reticulum Membranes (MAMs) and their prospective roles in kidney disease. Oxid Med Cell Longev. 2020;2020:3120539. doi: 10.1155/2020/3120539 32952849 10.1155/2020/3120539 PMC7487091

[123] Chen J Zhang H Yi X Dou Q Yang X He Y Chen J Chen K. Cellular senescence of renal tubular epithelial cells in acute kidney injury. Cell Death Discov. 2024;10:62. doi: 10.1038/s41420-024-01831-9 38316761 10.1038/s41420-024-01831-9 PMC10844256

[124] Bonventre JV Yang L. Cellular pathophysiology of ischemic acute kidney injury. J Clin Invest. 2011;121:4210–4221. doi: 10.1172/JCI45161 22045571 10.1172/JCI45161 PMC3204829

[125] Amor C Feucht J Leibold J Ho YJ Zhu C Alonso-Curbelo D Mansilla-Soto J Boyer JA Li X Giavridis T. Senolytic CAR T cells reverse senescence-associated pathologies. Nature. 2020;583:127–132. doi: 10.1038/s41586-020-2403-9 32555459 10.1038/s41586-020-2403-9 PMC7583560

[126] Amor C Fernandez-Maestre I Chowdhury S Ho YJ Nadella S Graham C Carrasco SE Nnuji-John E Feucht J Hinterleitner C. Prophylactic and long-lasting efficacy of senolytic CAR T cells against age-related metabolic dysfunction. Nat Aging. 2024;4:336–349. doi: 10.1038/s43587-023-00560-5 38267706 10.1038/s43587-023-00560-5 PMC10950785

[127] Matheus AS Tannus LR Cobas RA Palma CC Negrato CA Gomes MB. Impact of diabetes on cardiovascular disease: an update. Int J Hypertens. 2013;2013:653789. doi: 10.1155/2013/653789 23533715 10.1155/2013/653789 PMC3603160

[128] Guria S Hoory A Das S Chattopadhyay D Mukherjee S. Adipose tissue macrophages and their role in obesity-associated insulin resistance: an overview of the complex dynamics at play. Biosci Rep. 2023;43:BSR20220200. doi: 10.1042/BSR20220200 36718668 10.1042/BSR20220200 PMC10011338

[129] Lefere S Tacke F. Macrophages in obesity and non-alcoholic fatty liver disease: Crosstalk with metabolism. JHEP Rep. 2019;1:30–43. doi: 10.1016/j.jhepr.2019.02.004 32149275 10.1016/j.jhepr.2019.02.004 PMC7052781

[130] Rotbain Curovic V Theilade S Winther SA Tofte N Eugen-Olsen J Persson F Hansen TW Jeppesen J Rossing P. Soluble urokinase plasminogen activator receptor predicts cardiovascular events, kidney function decline, and mortality in patients with type 1 diabetes. Diabetes Care. 2019;42:1112–1119. doi: 10.2337/dc18-1427 30885954 10.2337/dc18-1427

[131] Guthoff M Wagner R Randrianarisoa E Hatziagelaki E Peter A Haring HU Fritsche A Heyne N. Soluble Urokinase Receptor (suPAR) predicts microalbuminuria in patients at risk for type 2 diabetes mellitus. Sci Rep. 2017;7:40627. doi: 10.1038/srep40627 28091558 10.1038/srep40627 PMC5238426

[132] Grynberg K Tian L Tesch G Ozols E Mulley WR Nikolic-Paterson DJ Ma FY. Mice with established diabetes show increased susceptibility to renal ischemia/reperfusion injury: protection by blockade of Jnk or Syk signaling pathways. Am J Pathol. 2022;192:441–453. doi: 10.1016/j.ajpath.2021.12.003 34954209 10.1016/j.ajpath.2021.12.003

[133] Yuan C Guo Z Yu S Jiang L Huang M. Development of inhibitors for uPAR: blocking the interaction of uPAR with its partners. Drug Discov Today. 2021;26:1076–1085. doi: 10.1016/j.drudis.2021.01.016 33486111 10.1016/j.drudis.2021.01.016

[134] Lin H Xu L Yu S Hong W Huang M Xu P. Therapeutics targeting the fibrinolytic system. Exp Mol Med. 2020;52:367–379. doi: 10.1038/s12276-020-0397-x 32152451 10.1038/s12276-020-0397-x PMC7156416

[135] Mattinzoli D Ikehata M D’Alonzo D De Fenza M Diana A Li M Armelloni S Tosoni A Del Gobbo A Collino F. Potential targeting of urokinase-type plasminogen activator receptor-formyl peptide receptor signaling to prevent recurrence in posttransplant primary podocytopathies. Am J Transplant. 2025;25:2104–2113. doi: 10.1016/j.ajt.2025.06.010 40517817 10.1016/j.ajt.2025.06.010

[136] Dal Monte M Cammalleri M Pecci V Carmosino M Procino G Pini A De Rosa M Pavone V Svelto M Bagnoli P. Inhibiting the urokinase-type plasminogen activator receptor system recovers STZ-induced diabetic nephropathy. J Cell Mol Med. 2019;23:1034–1049. doi: 10.1111/jcmm.14004 30426662 10.1111/jcmm.14004 PMC6349167

[137] Carriero MV Bifulco K Minopoli M Lista L Maglio O Mele L Di Carluccio G De Rosa M Pavone V. UPARANT. A urokinase receptor-derived peptide inhibitor of VEGF-driven angiogenesis with enhanced stability and in vitro and in vivo potency. Mol Cancer Ther. 2014;13:1092–1104. doi: 10.1158/1535-7163.MCT-13-0949 24705350 10.1158/1535-7163.MCT-13-0949

[138] De Fenza M Locri F Plastino F Chino M Maglio O Leone L Gazzaroli G Belleri M Giacomini A Kvanta A. Turn-adopting peptidomimetic as a formyl peptide receptor-1 antagonist. ACS Pharmacol Transl Sci. 2024;7:3476–3487. doi: 10.1021/acsptsci.4c00366 39539264 10.1021/acsptsci.4c00366 PMC11555506

[139] Cammalleri M Dal Monte M Locri F Pecci V De Rosa M Pavone V Bagnoli P. The urokinase-type plasminogen activator system as drug target in retinitis pigmentosa: New pre-clinical evidence in the rd10 mouse model. J Cell Mol Med. 2019;23:5176–5192. doi: 10.1111/jcmm.14391 31251468 10.1111/jcmm.14391 PMC6653070

[140] Childs BG Baker DJ Wijshake T Conover CA Campisi J van Deursen JM. Senescent intimal foam cells are deleterious at all stages of atherosclerosis. Science. 2016;354:472–477. doi: 10.1126/science.aaf6659 27789842 10.1126/science.aaf6659 PMC5112585

[141] Baker DJ Childs BG Durik M Wijers ME Sieben CJ Zhong J Saltness RA Jeganathan KB Verzosa GC Pezeshki A. Naturally occurring p16(Ink4a)-positive cells shorten healthy lifespan. Nature. 2016;530:184–189. doi: 10.1038/nature16932 26840489 10.1038/nature16932 PMC4845101

[142] Eskiocak O Gewolb J Shah V Rouse JA Chowdhury S Akyildiz EO Fernandez-Maestre I Boyer JA Filliol A Harris AS. Anti-uPAR CAR T cells reverse and prevent aging-associated defects in intestinal regeneration and fitness. Nat Aging. 2026;6:108–126. doi: 10.1038/s43587-025-01022-w 41291258 10.1038/s43587-025-01022-w PMC12823409

[143] Ma S Nakamura Y Hisaoka-Nakashima K Morioka N. Blockade of receptor for advanced glycation end-products with azeliragon ameliorates streptozotocin-induced diabetic neuropathy. Neurochem Int. 2023;163:105470. doi: 10.1016/j.neuint.2022.105470 36581174 10.1016/j.neuint.2022.105470

[144] Liu Y Shen W Chen Q Cao Q Di W Lan R Chen Z Bai J Han Z Xu W. Inhibition of RAGE by FPS-ZM1 alleviates renal injury in spontaneously hypertensive rats. Eur J Pharmacol. 2020;882:173228. doi: 10.1016/j.ejphar.2020.173228 32502492 10.1016/j.ejphar.2020.173228

[145] Myint KM Yamamoto Y Doi T Kato I Harashima A Yonekura H Watanabe T Shinohara H Takeuchi M Tsuneyama K. RAGE control of diabetic nephropathy in a mouse model: effects of RAGE gene disruption and administration of low-molecular weight heparin. Diabetes. 2006;55:2510–2522. doi: 10.2337/db06-0221 16936199 10.2337/db06-0221

[146] Bedard K Krause KH. The NOX family of ROS-generating NADPH oxidases: physiology and pathophysiology. Physiol Rev. 2007;87:245–313. doi: 10.1152/physrev.00044.2005 17237347 10.1152/physrev.00044.2005

[147] Juric M Rawat V Amaradhi R Zielonka J Ganesh T. Novel NADPH oxidase-2 inhibitors as potential anti-inflammatory and neuroprotective agents. Antioxidants (Basel). 2023;12:1660. doi: 10.3390/antiox12091660 37759963 10.3390/antiox12091660 PMC10525516

[148] Araujo J Logothetis CD. A potent SRC inhibitor in clinical development for the treatment of solid tumors. Cancer Treat Rev. 2010;36:492–500. doi: 10.1016/j.ctrv.2010.02.015 20226597 10.1016/j.ctrv.2010.02.015 PMC3940067

[149] Gerber DE Camidge DR Morgensztern D Cetnar J Kelly RJ Ramalingam SS Spigel DR Jeong W Scaglioni PP Zhang S. Phase 2 study of the focal adhesion kinase inhibitor defactinib (VS-6063) in previously treated advanced KRAS mutant non-small cell lung cancer. Lung Cancer. 2020;139:60–67. doi: 10.1016/j.lungcan.2019.10.033 31739184 10.1016/j.lungcan.2019.10.033 PMC6942685

[150] Trachtman H Kretzler M Gesualdo L Cross N Workeneh B Kaufeld J Meijers B Ye Z Chen Q Derebail VK. TRPC6 inhibition for the treatment of focal segmental glomerulosclerosis: a randomised, placebo-controlled, phase 2 trial of BI 764198. Lancet. 2026;407:587–598. doi: 10.1016/S0140-6736(25)02255-X 41616795 10.1016/S0140-6736(25)02255-X

[151] Zhou L Wang X Ren L Wang Y Liu X Wu K Gao Y Sun Y Lin X Hao H. TRPC6 inhibition by Z3571: A structure-based strategy to ameliorate glomerular and tubular dysfunction in chronic kidney disease. Biochem Pharmacol. 2026;243:117557. doi: 10.1016/j.bcp.2025.117557 41276089 10.1016/j.bcp.2025.117557

[152] Numaga-Tomita T Shimauchi T Oda S Tanaka T Nishiyama K Nishimura A Birnbaumer L Mori Y Nishida M. TRPC6 regulates phenotypic switching of vascular smooth muscle cells through plasma membrane potential-dependent coupling with PTEN. FASEB J. 2019;33:9785–9796. doi: 10.1096/fj.201802811R 31162976 10.1096/fj.201802811R PMC6704458

